# Nutrition-wide association study of microbiome diversity and composition in colorectal cancer patients

**DOI:** 10.1186/s12885-022-09735-6

**Published:** 2022-06-14

**Authors:** Tung Hoang, Min Jung Kim, Ji Won Park, Seung-Yong Jeong, Jeeyoo Lee, Aesun Shin

**Affiliations:** 1 Department of Preventive Medicine, Seoul National University College of Medicine, Seoul, 03080 South Korea; 2 Integrated Major in Innovative Medical Science, Seoul National University College of Medicine, Seoul, 03080 South Korea; 3 Department of Surgery, Seoul National University College of Medicine, Seoul, 03080 South Korea; 4 Cancer Research Institute, Seoul National University, Seoul, 03080 South Korea

**Keywords:** Nutrition-wide association study, Dietary intake, Gut microbiota, Colorectal cancer patients, Korean population

## Abstract

**Background:**

The effects of diet on the interaction between microbes and host health have been widely studied. However, its effects on the gut microbiota of patients with colorectal cancer (CRC) have not been elucidated. This study aimed to investigate the association between diet and the overall diversity and different taxa levels of the gut microbiota in CRC patients via the nutrition-wide association approach.

**Methods:**

This hospital-based study utilized data of 115 CRC patients who underwent CRC surgery in Department of Surgery, Seoul National University Hospital. Spearman correlation analyses were conducted for 216 dietary features and three alpha-diversity indices, *Firmicutes*/*Bacteroidetes* ratio, and relative abundance of 439 gut microbial taxonomy. To identify main enterotypes of the gut microbiota, we performed the principal coordinate analysis based on the β-diversity index. Finally, we performed linear regression to examine the association between dietary intake and main microbiome features, and linear discriminant analysis effect size (LEfSe) to identify bacterial taxa phylogenetically enriched in the low and high diet consumption groups.

**Results:**

Several bacteria were enriched in patients with higher consumption of mature pumpkin/pumpkin juice (ρ, 0.31 to 0.41) but lower intake of eggs (ρ, -0.32 to -0.26). We observed negative correlations between *Bacteroides fragilis* abundance and intake of pork (belly), beef soup with vegetables, animal fat, and fatty acids (ρ, -0.34 to -0.27); an inverse correlation was also observed between *Clostridium symbiosum* abundance and intake of some fatty acids, amines, and amino acids (ρ, -0.30 to -0.24). Furthermore, high intake of seaweed was associated with a 6% (95% CI, 2% to 11%) and 7% (95% CI, 2% to 11%) lower abundance of *Rikenellaceae* and *Alistipes*, respectively, whereas overall beverage consumption was associated with an 10% (95% CI, 2% to 18%) higher abundance of *Bacteroidetes*, *Bacteroidia*, and *Bacteroidales*, compared to that in the low intake group. LEfSe analysis identified phylogenetically enriched taxa associated with the intake of sugars and sweets, legumes, mushrooms, eggs, oils and fats, plant fat, carbohydrates, and monounsaturated fatty acids.

**Conclusions:**

Our data elucidates the diet-microbe interactions in CRC patients. Additional research is needed to understand the significance of these results in CRC prognosis.

**Supplementary Information:**

The online version contains supplementary material available at 10.1186/s12885-022-09735-6.

## Introduction

The gut microbiota of humans is a complex community comprising bacteria, archaea, and eukarya, with approximately 100 trillion microorganisms [1, 2]. It can interact with the host through several physiological processes, such as gut integrity consolidation, intestinal epithelium shaping, food digestion and energy metabolism, pathogen protection, and host immunity regulation [1–3]. Microbes begin colonizing the human gut immediately after birth; the gut microbiota community rapidly develops until the age of 3 years, gradually diversifies until the age of 40 years, and remains stable thereafter [4, 5]. However, the abundance and diversity of the gut microbiome are affected not only by host genetics but also by the health conditions of the host, such as inflammation, metabolic diseases, and cancer [3, 6–8].

In colorectal cancer (CRC) patients, tumorigenesis may alter the surrounding microenvironment, facilitate microbial translocation from the lumen to the lamina, and enhance the proliferation of opportunistic bacteria [9–11]. Overabundance of genera *Prevotella*, *Fusobacterium*, *Parvimonas*, *Porphyromonas*, *Peptostreptococcus*, *Bacteroides*, and *Gemella* has been observed in CRC patients compared to that in healthy individuals [9]. Even after CRC surgery, the composition of the gut microbiota varies between those with newly developed adenoma (similar to the gut microbiota of CRC patients) and those with a clean intestine (similar to the gut microbiota of healthy individuals) [12].

In the gastrointestinal tract, the microbiota plays a vital role in the fermentation of non-digestible components, especially the production of short-chain fatty acids (SCFAs), including acetate (central appetite regulation), propionate (gluconeogenesis and satiety signaling regulation), and butyrate (main energy source for human colonocytes) [13]. Numerous studies have demonstrated the effects of various dietary features on the gut microbiota. In general, a Western diet can inhibit mucus production and activate the penetrability of the colonic mucus barrier, which co-occurs with a shift toward a microbial community characterized by lower production of SCFAs due to fiber deficiency [14]. In contrast, individuals with the more consumption of fiber, which is highly contained in a plant-based diet, show a more diverse and stable microbial community and an increased abundance of SCFA-producing and lactic acid bacteria [14]. Individuals on polyphenol-based diets, another plant-based diet, have shown high abundance of *Bifidobacterium* and *Lactobacillus*, which have anti-pathogenic and anti-inflammatory effects [15]. Given the geographical variation in both the food culture and the microbiota structure [16, 17], recent studies have been conducted to elucidate the diet–microbiome relationship in the Korean population [18, 19]. However, to the best of our knowledge, the relationship between the diet and the microbiota, especially the effect of diet on CRC prognosis-related microbiota in CRC patients, has not been studied.

Therefore, in the present study, we performed a nutrition-wide association study to elucidate the effect of different dietary features on microbiome diversity and composition to evaluate the diet–microbiome association in CRC patients. Understanding the microbial response to diet in CRC patients is an important step for the development of therapeutic strategies based on dietary interventions to prevent the recurrence and improve the prognosis of CRC.

## Methods

### Participants and data collection

This hospital-based study utilized data from CRC patients who underwent CRC surgery in the Department of Surgery, Seoul National University Hospital, Seoul, Korea. General information on demographics (age and sex), family history of CRC, lifestyle (smoking status and alcohol consumption), and medical history (American Joint Committee on Cancer [AJCC] stage, neoadjuvant therapy and underlying chronic diseases) was collected. The height and weight of the participants were measured using a GL-310C (G-Tech International, Korea) machine. Body mass index (BMI) was calculated as weight (kg)/[height (m)]^2^. The patients collected their single fecal sample at one time point before the operation date (median, 5 days; interquartile range, 3–8 days), using a kit that was provided. Of patients who were indicated with a CRC resection between October 2017 and August 2019, informed consents and fecal specimens were obtained. A total of 331 patients remained after excluding those who could not be analyzed due to the absence or small amount of fecal sample prior to the surgery. Among them, dietary data were available for 115 subjects, who were included in the final analysis.

### Diet consumption

Dietary intake (g/day) was assessed using a validated semi-quantitative food frequency questionnaire (semi-quantitative FFQ) [20]. The average frequency of servings and the average portion size of 106 food items were recorded to estimate the average weight of and energy intake from food items consumed during the previous year. Daily intake of 106 food items, 663 food subitems, and 92 nutrients was calculated using the Computer-Aided Nutritional Analysis Program (CAN-Pro) 4.0 (Computer-Aided Nutritional Analysis Program, The Korean Nutrition Society, Seoul, Korea). The consumption of 663 subitems was then classified into 16 groups based on the nutrient profiles and culinary usage of each food item. Additionally, a residual method was used for the energy adjustment of the 106 food items, 16 food groups, and 92 nutrients [21]. Furthermore, we derived dietary patterns using principal component analysis (PCA). We constructed a scree plot to represent the variability of food groups based on dietary patterns. Food groups with factor loadings ≥ 0.20 were considered to have dominant contributions to the distinctive dietary pattern [22]. Moreover, we applied k-means clustering analysis based on the scores of the first two dietary patterns in the ‘factoextra’ package [23] to divide study participants into different groups based on dietary scores of the first two principal components.

### DNA extraction and 16S rRNA gene sequencing

DNA was isolated from fecal samples using the DNeasy power soil kit (Qiagen, Hilden, Germany) and quantified using the Quant-IT PicoGreen kit (Invitrogen), according to the manufacturer’s instructions. The genetic sequencing was performed after a median of 24 days (interquartile range, 16–41 days) from the date of sample collection. Sequencing libraries were prepared according to the Illumina 16S metagenomic sequencing library protocols to amplify the V3 and V4 regions of the 16S rRNA gene of bacteria. The universal primer pair with Illumina adapter overhang sequences used for the first amplification were as follows:

V3-forward primer:

5’-TCGTCGGCAGCGTCAGATGTGTATAAGAGACAGCCTACGGGNGGCWGCAG-3’

V4-reverse primer:

5’- GTCTCGTGGGCTCGGAGATGTGTATAAGAGACAGGACTACHVGGGTATCTAATCC-3’

After the sequencing process was completed for the MiSeq raw data, a FASTQ file was created using the MiSeq control software v2.2 and bcl2fastq (v1.8.4), and the PhiX sequence was removed using BWA. Paired-end data separated by each sample were assembled into one sequence using FLASH (1.2.11). After removing low-quality sequences, ambiguous sequences, and chimera sequences, which were considered as sequencing errors in the CD-HIT-OTU program, reads were clustered into operational taxonomic units (OTUs). A threshold of 97% was used to identify 16S rRNA sequence similarity within a species.

For the representative sequence of each OTU, BLASTN (v2.4.0) was performed using the nucleotide sequences present in the Reference database (NCBI 16S Microbial), and taxonomic assignment was performed using the sequence with the highest similarity; if the query coverage of the best hit matching the sequence from the database was less than 85% or the identity of the matched area was less than 85%, taxonomy was not defined. A comparative analysis of various microbial communities was performed using QIIME (v1.8) as the OTU abundance and taxonomic information.

### Statistical analysis

#### Descriptive statistics

To examine the distribution of demographics and lifestyles between the low fruit-vegetable and high fruit/low meat-poultry dietary groups, the Wilcoxon and chi-square tests were applied for continuous and categorical variables, respectively.

#### Microbial diversity and relative abundance

Rare species with mean relative abundances lower than 1 × 10^−6^ and/or unspecified phylum/class/order/family/genus were excluded during the construction of the phylogenetic tree. To examine within-sample diversity, we used the ‘vegan’ package and calculated α-diversity indices, including Chao1, Shannon, and Simpson indices, which represent the richness, evenness, and both the richness and evenness of the microbial community, respectively [24].

To identify the main enterotypes of the gut microbiota, we performed the principal coordinate analysis (PCoA) based on the β-diversity index calculated using the Jensen-Shannon divergence distance algorithm, which may be more efficient in capturing compositional changes with low-abundance factors and can work more stable than the Euclidean, Manhattan, hypersphere, and Aitchison-based distance measures [25], and then divided study participants into distinct enterotypes using the k-medoids method in the ‘cluster’ package [26]. For elucidating the microbial composition, zero values in microbial data were imputed using a compositional approach of the Bayesian-Multiplicative replacement using the ‘zCompositions’ package. The abundance data were then converted into the proportion form (relative abundance) [27, 28]. Additionally, given that the compositional data points did not map to the Euclidean space, but mapped to the Aitchison simplex, the transform compositions were converted into real space using a log-ratio transformation [27].

#### Microbial network structure

The network structure for pairwise correlations of the microbial community was constructed using the Gaussian graphical model (GGM) approach. In the GGM, missing edges between two nodes represent the conditional independence between these nodes, conditionalizing the remaining nodes [29, 30]. The pairwise correlation network structure was estimated using the lasso regularization method from the ‘glasso’ package to retain more solid edges only [31]. In general, increasing the level of regularization (lambda/tuning parameter) shifts the coefficient estimates of one node in correlation with the remaining nodes toward zero, leading to a sparser network. The optimal regulation parameter was selected from the cross-validation process of glasso in the ‘nethet’ package to ensure both the screening and sparsity assumptions of the network [32, 33].

#### Correlation analysis

The Spearman correlation coefficients between different dietary features and the microbial composition and diversity were calculated using the nutrition-wide association approach.

#### Association analysis

Based on the consumption of two diets, 16 food groups, six macronutrients, and three fatty acids, the participants were categorized into low intake and high intake groups based on the median value of consumption. Their associations with the relative abundance of major enterotypes, *Firmicutes*/*Bacteroidetes* (F/B) ratio, and α-diversity indices were investigated using multivariable regression analysis after adjustment for age, sex, family history of CRC, neoadjuvant therapy, smoking status, alcohol consumption, BMI, AJCC stage, and comorbidity.

Furthermore, we implemented a linear discriminant analysis (LDA) of effect size (LEfSe) in the Galaxy server to identify taxa (for all phylum, class, order, family, genus, and species levels) that significantly differed by consumption status. Differences were evaluated using a threshold for the logarithmic LDA score for discriminating features of 2.0 and *p*-values for the Wilcoxon test of 0.01.

## Results

### Identification of dietary patterns

We identified two main dietary patterns using PCA, which explained 69.5% of the variability of all food groups (Additional File 1: Figure S1). Considering factor loadings ≥ 0.20 to have dominant contributions to the distinctive dietary pattern, the low fruit-vegetable pattern was characterized by high intake of cereals and grains (0.64) and low intake of vegetables (-0.30) and fruits (-0.69), whereas the more healthy pattern was characterized by high intake of cereals and grains (0.62), and fruits (0.68) and low intake of meat and poultry (-0.25) (Table 1). After applying k-means clustering analysis, the study participants were divided into two groups based on diet: the low fruit-vegetable (*N* = 94) and high fruit/low meat-poultry (*N* = 21) groups.

**Table 1:** Factor loading matrix for first two dietary patterns

Food groups	Low fruit-vegetable	High fruit/low meat-poultry
Cereals and grains	0.64	0.62
Potatoes and starches	-0.03	-0.04
Sugars and sweets	0	0
Legumes	-0.11	-0.02
Seeds and nuts	0	0
Vegetables	-0.30	-0.18
Mushrooms	-0.01	-0.01
Fruits	-0.69	0.68
Meat and poultry	-0.01	-0.25
Eggs	-0.01	-0.03
Fish and shellfish	-0.02	-0.03
Seaweed	0	0
Milk and dairy	-0.1	-0.12
Oils and fats	0	-0.01
Beverages	-0.03	-0.19
Seasonings	-0.02	0
Others	0	0

### Characteristics of study participants according to dietary pattern groups

#### General characteristics

The distribution of demographics, lifestyle, and disease status of the study participants is presented in Table 2. The mean age of the 115 CRC patients was 60.8 years (standard deviation = 11.8), and 74 patients (64.3%) were men. At the time of enrollment, 105 (91.3%) participants had no family history of CRC, 92 (80.0%) were categorized as nonsmokers, and 74 (64.3%) as nondrinkers. Most of the study participants had no history of heart disease (*N* = 106, 92.2%), hypertension (*N* = 75, 65.2%), diabetes (*N* = 94, 81.7%), pulmonary disease (*N* = 106, 92.2%), and liver disease (*N* = 110, 95.7%). Overall, 67 patients (58.3%) had underlying diseases. Considering the dietary pattern groups, the participants in the low fruit-vegetable dietary group had a higher BMI than those in the high fruit/low meat-poultry dietary group (*p* = 0.02). Daily diet consumption of all study participants is shown in Additional file 2: Table S1.

**Table 2:** General characteristics of study participants

Factor	Low fruit-vegetable (*N* = 94)	High fruit/low meat-poultry (*N* = 21)	*P*-value	Total (*N* = 115)
**Age (mean ± sd, years)**	60.6 ± 11.9	62.0 ± 11.4	0.73	60.8 ± 11.8
≤ 50	20 (21.3)	3 (14.3)	0.91	23 (20.0)
51–60	25 (26.6)	6 (28.6)		31 (27.0)
61–70	29 (30.9)	7 (33.3)		36 (31.3)
≥ 71	20 (21.3)	5 (23.8)		25 (21.7)
**Sex**			> 0.99	
Female	34 (36.2)	7 (33.3)		41 (35.7)
Male	60 (63.8)	14 (66.7)		74 (64.3)
**Family of CRC**				
No	84 (89.4)	21 (100.0)	0.29	105 (91.3)
1st degree	7 (7.4)	0 (0.0)		7 (6.1)
2nd degree	3 (3.2)	0 (0.0)		3 (2.6)
**Smoking status**				
Nonsmoker	74 (78.7)	18 (85.7)	0.61	92 (80.0)
Former smoker	3 (3.2)	1 (4.8)		4 (3.5)
Current smoker	17 (18.1)	2 (9.5)		19 (16.5)
**Alcohol consumption**				
Nondrinker	63 (67.0)	11 (52.4)	0.37	74 (64.3)
Former drinker	1 (1.1)	0 (0.0)		1 (0.9)
Current drinker	30 (31.9)	10 (47.6)		40 (34.8)
**BMI (mean ± sd, kg/m**^**2**^**)**	24.9 ± 3.4	23.1 ± 2.4	0.02	24.6 ± 3.3
Normal (< = 22.9)	29 (30.9)	9 (42.9)	0.17	38 (33.0)
Overweight (23- ≤ 24.9)	17 (18.1)	6 (28.6)		23 (20.0)
Obesity (≥ 25.0)	48 (51.1)	6 (28.6)		54 (47.0)
**AJCC stage**				
0	4 (4.3)	1 (4.8)	0.54	5 (4.3)
1	18 (19.1)	1 (4.8)		19 (16.5)
2	27 (28.7)	7 (33.3)		34 (29.6)
3	29 (30.9)	9 (42.9)		38 (33.0)
4	16 (17.0)	3 (14.3)		19 (16.5)
**Neoadjuvant therapy**				
No	85 (90.4)	19 (90.5)	0.86	104 (90.4)
Chemotherapy and radiotherapy	6 (6.4)	1 (4.8)		7 (6.1)
Chemotherapy only	2 (2.1)	1 (4.8)		3 (2.6)
Radiotherapy only	1 (1.1)	0 (0.0)		1 (0.1)
**Comorbidity**				
No	38 (40.4)	10 (47.6)	0.72	48(41.7)
Yes	56 (59.6)	11 (52.4)		67 (58.3)
**Heart disease**				
No	86 (91.5)	20 (95.2)	0.90	106 (92.2)
Yes	8 (8.5)	1 (4.8)		9 (7.8)
**Hypertension**				
No	59 (62.8)	16 (76.2)	0.36	75 (65.2)
Yes	35 (37.2)	5 (23.8)		40 (34.8)
**Diabetes**				
No	75 (79.8)	19 (90.5)	0.40	94 (81.7)
Yes	19 (20.2)	2 (9.5)		21 (18.3)
**Pulmonary disease**				
No	87 (92.6)	19 (90.5)	> 0.99	106 (92.2)
Yes	7 (7.4)	2 (9.5)		9 (7.8)
**Liver disease**				
No	90 (95.7)	20 (95.2)	> 0.99	110 (95.7)
Yes	4 (4.3)	1 (4.8)		5 (4.3)
**Total energy (mean ± sd, kcal/day)**	1,495.0 ± 428.1	1,485.6 ± 906.4	0.96	1,493.3 ± 541.9

Data are presented as mean ± sd for continuous variables and count (percentage) for categorical variables. *P*-values are calculated from t-test for continuous variables and chi-square test for categorical variables

#### Microbial abundance according to dietary patterns

**Fig. 1:**
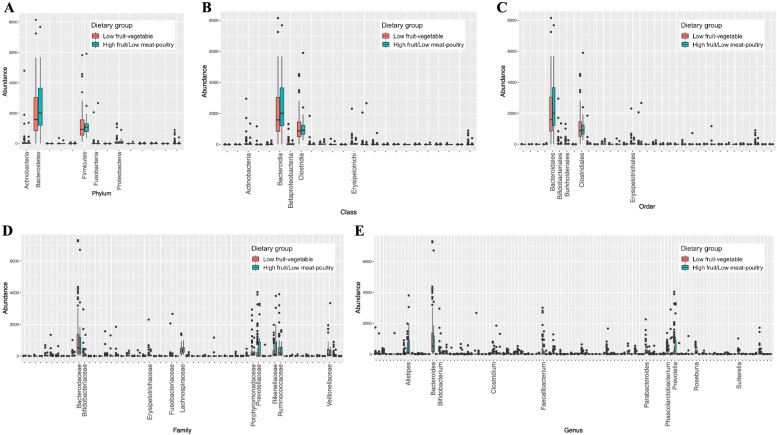
Distribution of (**A**) phylum, (**B**) class, (**C**) order, (**D**) family, and (**E**) genus abundance in low fruit-vegetable and high fruit/low meat-poultry dietary groups. X-axis shows top 5 most abundance taxa at phylum, class, and order levels, and top 10 most abundance taxa at family and genus levels

The abundance of microbial taxa at the phylum, class, order, family, genus, and species levels according to dietary pattern groups is shown in Fig. 1. In general, the microbial community appeared to be dominated by *Bacteroidetes* and *Firmicutes* at the phylum level, by *Bacteroidia* and *Clostridia* at the class level, and by *Bacteroidales* and *Clostridiales* at the order level. Additionally, the Wilcoxon test revealed a significant difference between the two groups in terms of the relative abundance of class *RF3* (*p* = 0.01), orders *ML615J-28* (*p* = 0.01), *RF32* (*p* = 0.03), and *Spirochaetales* (*p* = 0.04), families *RF16*, *S24-7*, and *Spirochaetaceae* (*p* = 0.04), and genera *Acidaminococcus*, *Anaerococcus*, *Butyrivibrio*, *Enterobacter*, *Megamonas*, and *Treponema* (*p* = 0.04).

#### Microbial network structure

Additional file 1: Figures S2-S6 and Additional file 2: Table S2 present differences in the interconnected relationship of the microbial community between the two diet groups. Considering the dominant taxa, *Bacteroidetes* was negatively correlated with *Actinobacteria* in the low fruit-vegetable dietary group, whereas the pairwise correlation was positive in the high fruit/low meat-poultry dietary group (Additional file 1: Figure S2). At the class level, *Bacteroidia* was negatively correlated with *Betaprobacteria* in the low fruit-vegetable dietary group, whereas the pairwise correlation was positive in the high fruit/low meat-poultry dietary group (Additional file 1: Figure S3). Nevertheless, given the higher tuning parameter, the GGM networks of microbial taxonomy for the high fruit/low meat-poultry dietary group were sparser than those for the low fruit-vegetable dietary group.

### Identification of main enterotypes in colorectal cancer patients

**Fig. 2:**
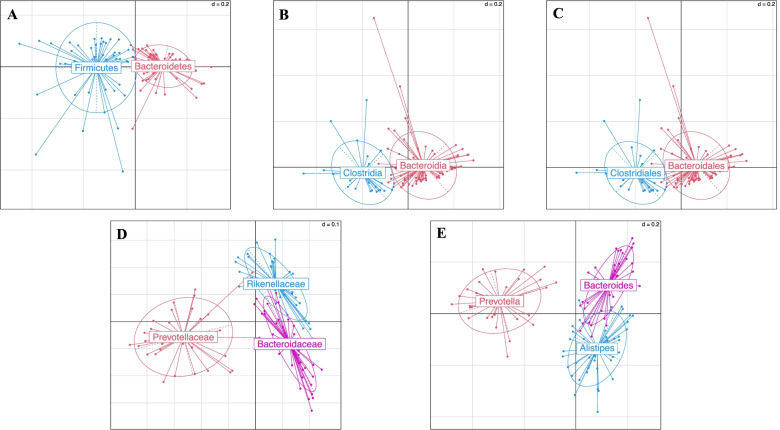
Classification of enterotypes at (**A**) phylum, (**B**) class, (**C**) order, (**D**) family, and (**E**) genus levels

**Fig. 3:**
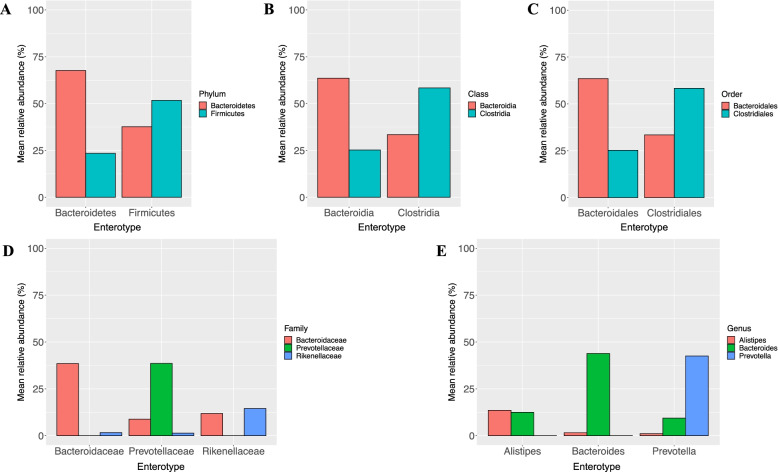
Mean relative abundance of major (**A**) phylum, (**B**) class, (**C**) order, (**D**) family, and (**E**) genus according to the enterotypes identified as dominant bacteria

Enterotypes of the fecal microbiota among CRC patients based on the Jensen-Shannon divergence distance algorithm are presented in Fig. 2. The enterotypes of the dominant bacteria and lower levels were gram-negative *Bacteroidetes* (*N* = 67, 58.3%) and gram-positive *Firmicutes* (*N* = 48, 41.7%) at the phylum level, *Bacteroidia* (*N* = 86, 74.8%) and *Clostridia* (*N* = 29, 25.2%) at the class level, *Bacteroidales* (*N* = 86, 74.8%) and *Clostridiales* (*N* = 29, 25.2%) at the order level, *Bacteroidaceae* (*N* = 44, 38.3%), *Prevotellaceae* (*N* = 32, 27.8%), and *Rikenellaceae* (*N* = 39, 33.9%) at the family level, and *Alistipes* (*N* = 44, 38.3%), *Bacteroides* (*N* = 41, 35.7%), and *Prevotella* (*N* = 30, 26.1%) at the genus level. The relative abundances of these dominant bacteria according to different taxonomy levels are shown in Fig. 3.

### Correlation of dietary intake with microbial diversity and composition

#### Diet and microbial alpha-diversity

Additional file 2: Table S1 displays the Spearman correlations of log-transformed alpha-diversity indices, including Chao1, Shannon, and Simpson indices, with continuous intake of 106 food items, 16 food groups, two dietary patterns, and 92 nutrients. Overall, we did not observe any significant correlations between within-sample diversity and diet consumption in CRC patients (false discovery rate [FDR], *p* > 0.05).

#### Diet and microbial composition

**Fig. 4:**
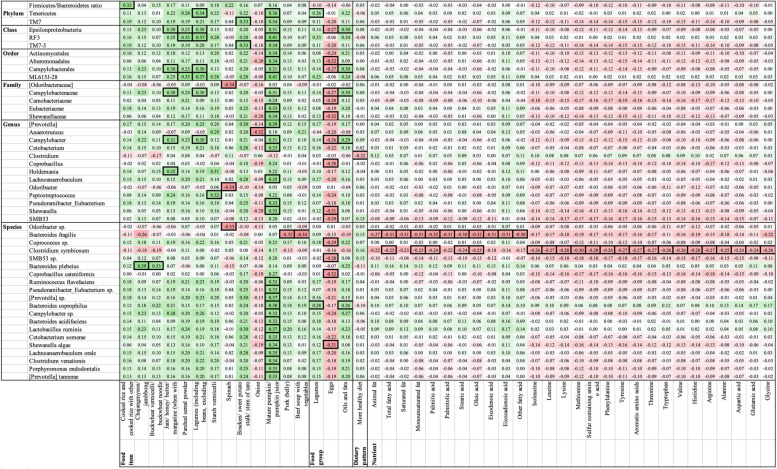
Heatmap for Spearman significant correlations between diet consumption and relative abundance of microbial taxonomy. Data are presented as Spearman correlation coefficients. Green color represents positive correlation and red color represents negative correlation. Outside borders indicate false discovery rate *p*-values are less than 0.05

Additional file 2: Table S3 displays the Spearman correlation coefficients of the relative abundance of different bacteria at all taxon levels with all dietary features. Crude and FDR *p*-values are presented in Additional file 2: Tables S4-S5, respectively. After adjusting for multiple comparisons, the F/B ratio and the abundance of 48 bacteria were significantly correlated with intakes of 45 diet features, with tight correlations (ρ from -0.34, -0.24, and from 0.23 to 0.41) (Fig. 4). This included the enrichment of several bacteria with higher consumption of mature pumpkin or pumpkin juice (ρ, 0.31 to 0.41) but lower intake of eggs (ρ, -0.32 to -0.26). There were significant negative correlations between the relative abundance of *Bacteroides fragilis* and consumption of pork (belly) (ρ, -0.32), beef soup with vegetables (ρ, -0.34), animal fat (ρ, -0.27), and fatty acids (ρ, -0.32 to -0.30). An inverse correlation was observed between the relative abundance of *Clostridium symbiosum* and intake of some fatty acids, amines, and amino acids (ρ, -0.30 to -0.24). In addition, the high fruit/low meat-poultry dietary pattern was inversely correlated only with the abundance of the genus *Clostridium*.

### Association of dietary consumption and microbial taxonomy

#### Linear regression model

Table 3 presents the beta coefficients and their corresponding 95% CIs for the difference in taxon diversity and relative abundance between low and high intake groups (food groups, macronutrients, and fatty acids). The linear regression model was used for log-transformation of the alpha-diversity indices, F/B ratio, and relative abundance of dominant bacteria. Participants with a high consumption of seaweed showed a significantly lower relative abundance of family *Rikenellaceae* (β, -0.06, 95% CI, -0.11 to -0.02) and genus *Alistipes* (β, -0.07, and 95% CI, -0.11 to -0.02) than those in the low intake group. These associations were persistent after adjustment for age, sex, family history of colorectal cancer, smoking status, alcohol consumption, BMI, AJCC stage, and underlying diseases. Additionally, the participants with a high intake of beverages had a 10% (95% CI, 2% to 18%) higher relative abundance of *Bacteroidetes*, *Bacteroidia*, and *Bacteroidales* than those with a low intake of beverages in the multivariable model.

**Table 3:** Linear regression coefficients and their 95% confidence intervals for the difference of microbial relative abundance and alpha diversity indices in high intake group compared to low intake group

Dietary feature	Chao1 ^a^	Shannon ^a^	Simpson ^a^	F/B ratio	Bacteroidetes	Firmicutes	Bacteroidia	Clostridia	Bacteroidales	Clostridiales	Bacteroidaceae	Rikenellaceae	Alistipes	Bacteroides
**Univariable model**														
**Food groups**														
Cereals and grains	0.08 (-0.06, 0.21)	0.06 (-0.05, 0.16)	0.05 (-0.04, 0.13)	-3.48 (-17.4, 10.4)	0.06 (-0.02, 0.14)	-0.06 (-0.13, 0.01)	0.06 (-0.02, 0.14)	-0.04 (-0.11, 0.03)	0.06 (-0.02, 0.14)	-0.04 (-0.11, 0.03)	0.01 (-0.07, 0.09)	0.02 (-0.03, 0.07)	0.02 (-0.03, 0.07)	0.01 (-0.08, 0.09)
Potatoes and starches	-0.09 (-0.22, 0.04)	-0.08 (-0.18, 0.03)	-0.05 (-0.14, 0.03)	2.71 (-11.2, 16.6)	0 (-0.08, 0.07)	-0.03 (-0.11, 0.04)	0 (-0.08, 0.07)	-0.05 (-0.12, 0.02)	0 (-0.08, 0.07)	-0.05 (-0.12, 0.02)	-0.01 (-0.09, 0.07)	-0.04 (-0.08, 0.01)	-0.04 (-0.08, 0.01)	-0.01 (-0.09, 0.07)
Sugars and sweets	0.07 (-0.06, 0.21)	-0.02 (-0.13, 0.09)	-0.01 (-0.10, 0.07)	-10.2 (-23.97, 3.64)	0.03 (-0.05, 0.11)	-0.07 (-0.14, 0.01)	0.03 (-0.05, 0.11)	-0.06 (-0.13, 0.01)	0.03 (-0.05, 0.11)	-0.06 (-0.13, 0.01)	-0.02 (-0.10, 0.06)	-0.03 (-0.08, 0.02)	-0.03 (-0.08, 0.02)	-0.02 (-0.10, 0.06)
Legumes	-0.03 (-0.16, 0.11)	-0.06 (-0.17, 0.04)	-0.04 (-0.13, 0.04)	3.08 (-10.84, 17)	-0.03 (-0.11, 0.05)	0 (-0.08, 0.07)	-0.03 (-0.11, 0.05)	-0.03 (-0.10, 0.04)	-0.03 (-0.11, 0.05)	-0.03 (-0.10, 0.04)	0.03 (-0.05, 0.11)	-0.02 (-0.07, 0.03)	-0.02 (-0.07, 0.03)	0.02 (-0.06, 0.11)
Seeds and nuts	0.1 (-0.03, 0.23)	0.1 (0, 0.21)	0.07 (-0.01, 0.15)	-9.68 (-23.5, 4.13)	-0.02 (-0.10, 0.06)	-0.01 (-0.09, 0.06)	-0.02 (-0.10, 0.06)	0 (-0.07, 0.07)	-0.02 (-0.10, 0.06)	0 (-0.07, 0.07)	0.02 (-0.06, 0.10)	0.01 (-0.04, 0.05)	0.01 (-0.04, 0.06)	0.03 (-0.06, 0.11)
Vegetables	-0.03 (-0.16, 0.10)	-0.02 (-0.12, 0.09)	-0.02 (-0.11, 0.06)	2.98 (-10.9, 16.9)	-0.04 (-0.12, 0.04)	0.03 (-0.05, 0.10)	-0.04 (-0.12, 0.04)	0.01 (-0.06, 0.08)	-0.04 (-0.12, 0.04)	0.01 (-0.06, 0.08)	0 (-0.08, 0.08)	-0.04 (-0.08, 0.01)	-0.04 (-0.08, 0.01)	0 (-0.08, 0.08)
Mushrooms	0.1 (-0.03, 0.23)	0.04 (-0.07, 0.14)	0.02 (-0.06, 0.10)	-9.46 (-23.3, 4.36)	0 (-0.08, 0.08)	0 (-0.08, 0.07)	0 (-0.08, 0.08)	0 (-0.07, 0.07)	0 (-0.08, 0.08)	0 (-0.07, 0.07)	0.03 (-0.05, 0.11)	0 (-0.05, 0.05)	0 (-0.05, 0.05)	0.03 (-0.05, 0.11)
Fruits	0.09 (-0.04, 0.22)	0.08 (-0.03, 0.18)	0.05 (-0.03, 0.14)	-9.57 (-23.4, 4.25)	0.01 (-0.07, 0.09)	-0.03 (-0.10, 0.04)	0.01 (-0.07, 0.09)	-0.02 (-0.09, 0.05)	0.01 (-0.07, 0.09)	-0.02 (-0.09, 0.05)	0.01 (-0.07, 0.09)	0.03 (-0.02, 0.07)	0.03 (-0.02, 0.07)	0.01 (-0.07, 0.09)
Meat and poultry	0.08 (-0.05, 0.21)	0.02 (-0.09, 0.13)	0 (-0.08, 0.08)	-9.32 (-23.1, 4.51)	0.02 (-0.06, 0.10)	-0.01 (-0.09, 0.06)	0.02 (-0.06, 0.10)	-0.01 (-0.08, 0.06)	0.02 (-0.06, 0.10)	-0.01 (-0.08, 0.06)	-0.02 (-0.10, 0.06)	0.05 (0, 0.10)	0.05 (0, 0.10)	-0.02 (-0.10, 0.06)
Eggs	0.06 (-0.08, 0.19)	0.02 (-0.09, 0.13)	0 (-0.08, 0.08)	-9.25 (-23.1, 4.58)	0.02 (-0.06, 0.10)	0.04 (-0.03, 0.12)	0.02 (-0.06, 0.10)	0.04 (-0.03, 0.11)	0.02 (-0.06, 0.10)	0.04 (-0.03, 0.11)	-0.02 (-0.10, 0.06)	-0.02 (-0.07, 0.02)	-0.02 (-0.07, 0.02)	-0.02 (-0.10, 0.06)
Fish and shellfish	0.03 (-0.10, 0.17)	-0.03 (-0.14, 0.07)	-0.05 (-0.13, 0.03)	-9.48 (-23.3, 4.34)	0.07 (-0.01, 0.15)	-0.05 (-0.12, 0.03)	0.07 (-0.01, 0.15)	-0.05 (-0.12, 0.02)	0.07 (-0.01, 0.15)	-0.05 (-0.12, 0.02)	0.08 (0, 0.16)	-0.02 (-0.07, 0.03)	-0.02 (-0.07, 0.03)	0.08 (0, 0.16)
Seaweed	-0.06 (-0.19, 0.07)	-0.07 (-0.17, 0.04)	-0.07 (-0.15, 0.02)	9.35 (-4.48, 23.2)	-0.03 (-0.11, 0.05)	0 (-0.07, 0.08)	-0.03 (-0.11, 0.05)	-0.01 (-0.08, 0.06)	-0.03 (-0.11, 0.05)	-0.01 (-0.08, 0.06)	0 (-0.08, 0.08)	**-0.06 (-0.11, -0.01)**	**-0.06 (-0.11, -0.02)**	0 (-0.08, 0.08)
Milk and dairy	-0.04 (-0.17, 0.09)	-0.08 (-0.19, 0.02)	-0.06 (-0.14, 0.02)	2.54 (-11.4, 16.5)	0.04 (-0.04, 0.12)	-0.01 (-0.08, 0.07)	0.04 (-0.04, 0.12)	-0.01 (-0.08, 0.06)	0.04 (-0.04, 0.12)	-0.01 (-0.08, 0.06)	0.04 (-0.04, 0.12)	-0.04 (-0.09, 0.01)	-0.04 (-0.09, 0.01)	0.04 (-0.04, 0.13)
Oils and fats	0.07 (-0.06, 0.20)	0.04 (-0.06, 0.15)	0.05 (-0.04, 0.13)	-9.72 (-23.5, 4.1)	-0.01 (-0.09, 0.07)	-0.02 (-0.10, 0.05)	-0.01 (-0.09, 0.07)	-0.02 (-0.09, 0.05)	-0.01 (-0.09, 0.07)	-0.02 (-0.09, 0.05)	-0.08 (-0.16, 0)	-0.01 (-0.06, 0.04)	-0.01 (-0.06, 0.04)	-0.08 (-0.16, 0)
Beverages	0.06 (-0.07, 0.20)	-0.01 (-0.12, 0.09)	-0.02 (-0.11, 0.06)	-9.71 (-23.5, 4.1)	0.08 (0, 0.16)	-0.05 (-0.12, 0.03)	0.08 (0, 0.16)	-0.03 (-0.10, 0.05)	0.08 (0, 0.16)	-0.03 (-0.10, 0.05)	0.02 (-0.06, 0.10)	0.01 (-0.04, 0.06)	0.01 (-0.04, 0.06)	0.03 (-0.06, 0.11)
Seasonings	0.08 (-0.06, 0.21)	0.06 (-0.04, 0.17)	0.04 (-0.04, 0.12)	-9.34 (-23.2, 4.48)	-0.02 (-0.10, 0.06)	-0.01 (-0.08, 0.07)	-0.02 (-0.10, 0.06)	-0.01 (-0.08, 0.06)	-0.02 (-0.10, 0.06)	-0.01 (-0.08, 0.06)	-0.07 (-0.14, 0.01)	0.05 (0, 0.09)	0.05 (0, 0.09)	-0.07 (-0.15, 0.01)
**Macronutrients**														
Plant protein	0.02 (-0.12, 0.15)	0.02 (-0.08, 0.13)	0.01 (-0.07, 0.10)	2.75 (-11.2, 16.7)	-0.04 (-0.12, 0.04)	-0.01 (-0.08, 0.07)	-0.04 (-0.12, 0.04)	-0.02 (-0.09, 0.05)	-0.04 (-0.12, 0.04)	-0.02 (-0.09, 0.05)	-0.03 (-0.11, 0.05)	-0.04 (-0.08, 0.01)	-0.04 (-0.09, 0.01)	-0.04 (-0.12, 0.04)
Animal protein	0.04 (-0.09, 0.18)	0.03 (-0.08, 0.14)	0.01 (-0.08, 0.09)	-9.07 (-22.9, 4.76)	-0.03 (-0.10, 0.05)	0.04 (-0.03, 0.12)	-0.03 (-0.10, 0.05)	0.04 (-0.03, 0.11)	-0.03 (-0.10, 0.05)	0.04 (-0.03, 0.11)	-0.04 (-0.12, 0.04)	0.01 (-0.03, 0.06)	0.01 (-0.03, 0.06)	-0.04 (-0.12, 0.04)
Plant fat	-0.02 (-0.15, 0.12)	0.01 (-0.10, 0.11)	0.01 (-0.07, 0.09)	-9.08 (-22.9, 4.75)	-0.03 (-0.11, 0.05)	0.02 (-0.06, 0.09)	-0.03 (-0.11, 0.05)	0.01 (-0.06, 0.08)	-0.03 (-0.11, 0.05)	0.01 (-0.06, 0.08)	-0.01 (-0.09, 0.07)	-0.02 (-0.07, 0.02)	-0.02 (-0.07, 0.02)	-0.01 (-0.10, 0.07)
Animal fat	0.04 (-0.09, 0.18)	0.03 (-0.08, 0.14)	0.01 (-0.07, 0.09)	-9.18 (-23.0, 4.65)	-0.01 (-0.08, 0.07)	0.03 (-0.05, 0.10)	-0.01 (-0.08, 0.07)	0.02 (-0.05, 0.09)	-0.01 (-0.08, 0.07)	0.02 (-0.05, 0.09)	-0.01 (-0.09, 0.07)	-0.01 (-0.06, 0.04)	-0.01 (-0.06, 0.04)	-0.01 (-0.09, 0.07)
Carbohydrates	-0.09 (-0.22, 0.04)	-0.06 (-0.16, 0.05)	-0.02 (-0.10, 0.06)	8.97 (-4.87, 22.8)	0.02 (-0.06, 0.10)	-0.03 (-0.10, 0.05)	0.02 (-0.06, 0.10)	-0.02 (-0.09, 0.05)	0.02 (-0.06, 0.10)	-0.02 (-0.09, 0.05)	0.02 (-0.06, 0.10)	-0.04 (-0.08, 0.01)	-0.04 (-0.08, 0.01)	0.02 (-0.06, 0.11)
Fiber	0.01 (-0.13, 0.14)	0.02 (-0.09, 0.12)	0 (-0.08, 0.08)	3.2 (-10.7, 17.1)	-0.08 (-0.16, 0)	0.05 (-0.02, 0.13)	-0.08 (-0.16, 0)	0.04 (-0.03, 0.11)	-0.08 (-0.16, 0)	0.04 (-0.03, 0.11)	-0.04 (-0.12, 0.04)	-0.03 (-0.07, 0.02)	-0.03 (-0.07, 0.02)	-0.04 (-0.12, 0.04)
**Fatty acids**														
SFAs	-0.03 (-0.16, 0.11)	-0.07 (-0.18, 0.04)	-0.07 (-0.15, 0.01)	-9.61 (-23.4, 4.21)	0.05 (-0.03, 0.13)	-0.02 (-0.09, 0.05)	0.05 (-0.03, 0.13)	-0.01 (-0.08, 0.06)	0.05 (-0.03, 0.13)	-0.01 (-0.08, 0.06)	0.02 (-0.06, 0.10)	-0.02 (-0.06, 0.03)	-0.02 (-0.06, 0.03)	0.02 (-0.06, 0.10)
MUFAs	-0.04 (-0.18, 0.09)	-0.06 (-0.16, 0.05)	-0.06 (-0.15, 0.02)	-9.49 (-23.3, 4.33)	0.02 (-0.06, 0.10)	0 (-0.08, 0.07)	0.02 (-0.06, 0.10)	0 (-0.07, 0.07)	0.02 (-0.06, 0.10)	0 (-0.07, 0.07)	0.02 (-0.06, 0.10)	0 (-0.04, 0.05)	0 (-0.04, 0.05)	0.03 (-0.06, 0.11)
PUFAs	0.07 (-0.06, 0.21)	0.05 (-0.06, 0.15)	0.01 (-0.07, 0.09)	-9.29 (-23.1, 4.53)	-0.03 (-0.10, 0.05)	0.06 (-0.02, 0.13)	-0.03 (-0.10, 0.05)	0.06 (-0.01, 0.13)	-0.03 (-0.10, 0.05)	0.06 (-0.01, 0.13)	-0.05 (-0.13, 0.03)	0 (-0.04, 0.05)	0 (-0.04, 0.05)	-0.05 (-0.13, 0.03)
**Multivariable model**														
**Food groups**														
Cereals and grains	0.08 (-0.07, 0.23)	0.06 (-0.05, 0.18)	0.05 (-0.04, 0.13)	-2.74 (-18.1, 12.6)	0.06 (-0.02, 0.15)	-0.07 (-0.15, 0.02)	0.06 (-0.02, 0.15)	-0.05 (-0.13, 0.03)	0.06 (-0.02, 0.15)	-0.05 (-0.13, 0.03)	-0.01 (-0.10, 0.08)	0.03 (-0.02, 0.08)	0.03 (-0.02, 0.08)	-0.01 (-0.1, 0.08)
Potatoes and starches	-0.09 (-0.24, 0.06)	-0.08 (-0.20, 0.04)	-0.05 (-0.14, 0.04)	1.33 (-14.4, 17.1)	-0.01 (-0.10, 0.07)	-0.03 (-0.11, 0.06)	-0.01 (-0.1, 0.07)	-0.05 (-0.13, 0.03)	-0.01 (-0.10, 0.07)	-0.05 (-0.13, 0.03)	-0.02 (-0.11, 0.07)	-0.01 (-0.06, 0.04)	-0.01 (-0.07, 0.04)	-0.02 (-0.11, 0.07)
Sugars and sweets	0.09 (-0.07, 0.24)	0.01 (-0.11, 0.13)	0.01 (-0.08, 0.10)	-7.6 (-23.5, 8.30)	0 (-0.09, 0.09)	-0.05 (-0.13, 0.04)	0 (-0.09, 0.09)	-0.03 (-0.12, 0.05)	0 (-0.09, 0.09)	-0.03 (-0.12, 0.05)	-0.03 (-0.12, 0.06)	-0.03 (-0.08, 0.03)	-0.03 (-0.08, 0.02)	-0.03 (-0.12, 0.06)
Legumes	-0.03 (-0.18, 0.11)	-0.06 (-0.17, 0.05)	-0.04 (-0.13, 0.04)	5.69 (-9.20, 20.5)	-0.04 (-0.12, 0.05)	0.01 (-0.07, 0.09)	-0.04 (-0.12, 0.05)	-0.02 (-0.1, 0.06)	-0.04 (-0.12, 0.05)	-0.02 (-0.1, 0.06)	0.01 (-0.08, 0.09)	-0.02 (-0.06, 0.03)	-0.02 (-0.07, 0.03)	0 (-0.08, 0.09)
Seeds and nuts	0.10 (-0.04, 0.25)	0.10 (-0.01, 0.21)	0.07 (-0.01, 0.16)	-10.6 (-25.5, 4.4)	-0.02 (-0.10, 0.07)	-0.02 (-0.1, 0.06)	-0.02 (-0.10, 0.07)	-0.01 (-0.08, 0.07)	-0.02 (-0.10, 0.07)	-0.01 (-0.08, 0.07)	0.02 (-0.06, 0.11)	0.01 (-0.04, 0.06)	0.01 (-0.04, 0.06)	0.03 (-0.06, 0.12)
Vegetables	-0.04 (-0.19, 0.11)	-0.04 (-0.15, 0.08)	-0.04 (-0.12, 0.05)	2.52 (-12.61, 17.7)	-0.03 (-0.11, 0.06)	0.02 (-0.07, 0.10)	-0.03 (-0.11, 0.06)	0 (-0.08, 0.08)	-0.03 (-0.11, 0.06)	0 (-0.08, 0.08)	0.02 (-0.07, 0.1)	-0.05 (-0.10, 0)	-0.05 (-0.10, 0)	0.02 (-0.07, 0.11)
Mushrooms	0.09 (-0.06, 0.24)	0.04 (-0.08, 0.15)	0.02 (-0.07, 0.11)	-10.1 (-25.3, 5.10)	0.01 (-0.08, 0.10)	-0.02 (-0.10, 0.07)	0.01 (-0.08, 0.10)	-0.01 (-0.08, 0.07)	0.01 (-0.08, 0.10)	-0.01 (-0.08, 0.07)	0.02 (-0.07, 0.11)	-0.01 (-0.06, 0.04)	-0.01 (-0.06, 0.04)	0.02 (-0.07, 0.11)
Fruits	0.10 (-0.05, 0.25)	0.09 (-0.02, 0.20)	0.07 (-0.02, 0.15)	-8.59 (-23.7, 6.50)	0.01 (-0.08, 0.09)	-0.03 (-0.11, 0.05)	0.01 (-0.08, 0.09)	-0.01 (-0.09, 0.06)	0.01 (-0.08, 0.09)	-0.01 (-0.09, 0.06)	0.03 (-0.06, 0.12)	0.04 (-0.01, 0.09)	0.04 (-0.01, 0.09)	0.03 (-0.06, 0.12)
Meat and poultry	0.11 (-0.05, 0.26)	0.01 (-0.11, 0.13)	-0.01 (-0.1, 0.09)	-10.0 (-26.1, 5.97)	0.03 (-0.06, 0.12)	-0.03 (-0.12, 0.05)	0.03 (-0.06, 0.12)	-0.02 (-0.11, 0.06)	0.03 (-0.06, 0.12)	-0.02 (-0.11, 0.06)	-0.01 (-0.1, 0.08)	0.03 (-0.02, 0.08)	0.03 (-0.02, 0.08)	-0.01 (-0.11, 0.08)
Eggs	0.07 (-0.08, 0.22)	0.02 (-0.1, 0.13)	0 (-0.09, 0.09)	-12.4 (-27.3, 2.49)	0.03 (-0.05, 0.12)	0.03 (-0.05, 0.11)	0.03 (-0.05, 0.12)	0.04 (-0.04, 0.11)	0.03 (-0.05, 0.12)	0.04 (-0.04, 0.11)	-0.02 (-0.11, 0.07)	-0.04 (-0.09, 0.01)	-0.04 (-0.09, 0.01)	-0.02 (-0.11, 0.07)
Fish and shellfish	0.03 (-0.12, 0.18)	-0.05 (-0.17, 0.06)	-0.07 (-0.16, 0.02)	-8.69 (-23.7, 6.34)	0.07 (-0.01, 0.16)	-0.06 (-0.14, 0.03)	0.07 (-0.01, 0.16)	-0.06 (-0.14, 0.02)	0.07 (-0.01, 0.16)	-0.06 (-0.13, 0.02)	0.10 (0.01, 0.18)	-0.02 (-0.07, 0.02)	-0.03 (-0.08, 0.02)	0.1 (0.01, 0.18)
Seaweed	-0.06 (-0.21, 0.09)	-0.06 (-0.17, 0.05)	-0.06 (-0.15, 0.03)	8.91 (-6.08, 23.9)	-0.05 (-0.14, 0.03)	0.01 (-0.07, 0.09)	-0.05 (-0.13, 0.03)	0 (-0.08, 0.08)	-0.05 (-0.13, 0.03)	0 (-0.08, 0.08)	0 (-0.09, 0.09)	**-0.06 (-0.11, -0.02)**	**-0.07 (-0.11, -0.02)**	0 (-0.09, 0.09)
Milk and dairy	-0.03 (-0.18, 0.11)	-0.07 (-0.18, 0.04)	-0.05 (-0.13, 0.04)	2.98 (-12.0, 17.9)	0.02 (-0.07, 0.10)	0 (-0.08, 0.09)	0.02 (-0.07, 0.10)	0 (-0.08, 0.07)	0.02 (-0.07, 0.10)	0 (-0.08, 0.07)	0.04 (-0.04, 0.13)	-0.04 (-0.08, 0.01)	-0.04 (-0.09, 0.01)	0.04 (-0.04, 0.13)
Oils and fats	0.05 (-0.11, 0.20)	0.03 (-0.09, 0.15)	0.04 (-0.05, 0.14)	-5.8 (-21.7, 10.1)	-0.04 (-0.13, 0.05)	-0.01 (-0.10, 0.08)	-0.04 (-0.13, 0.05)	-0.01 (-0.09, 0.07)	-0.04 (-0.13, 0.05)	-0.01 (-0.09, 0.07)	-0.08 (-0.17, 0.01)	-0.02 (-0.07, 0.03)	-0.02 (-0.07, 0.03)	-0.08 (-0.17, 0.01)
Beverages	0.07 (-0.07, 0.22)	-0.02 (-0.13, 0.09)	-0.03 (-0.12, 0.05)	-11.6 (-26.3, 3.10)	**0.10 (0.02, 0.18)**	-0.07 (-0.15, 0.01)	**0.10 (0.02, 0.18)**	-0.04 (-0.12, 0.04)	**0.10 (0.02, 0.18)**	-0.04 (-0.12, 0.04)	0.02 (-0.06, 0.11)	0 (-0.05, 0.05)	0 (-0.05, 0.05)	0.02 (-0.06, 0.11)
Seasonings	0.06 (-0.09, 0.21)	0.03 (-0.08, 0.14)	0.02 (-0.07, 0.11)	-9.03 (-24.2, 6.10)	-0.02 (-0.10, 0.07)	-0.02 (-0.10, 0.06)	-0.02 (-0.10, 0.07)	-0.02 (-0.1, 0.05)	-0.02 (-0.1, 0.07)	-0.02 (-0.10, 0.05)	-0.05 (-0.14, 0.03)	0.04 (-0.01, 0.08)	0.04 (-0.01, 0.09)	-0.06 (-0.14, 0.03)
**Macronutrients**														
Plant protein	0 (-0.15, 0.15)	0 (-0.11, 0.11)	-0.01 (-0.09, 0.08)	5.9 (-9.3, 21.0)	-0.03 (-0.12, 0.05)	-0.01 (-0.09, 0.07)	-0.03 (-0.12, 0.05)	-0.02 (-0.1, 0.05)	-0.03 (-0.12, 0.05)	-0.02 (-0.1, 0.05)	-0.04 (-0.12, 0.05)	-0.02 (-0.07, 0.03)	-0.02 (-0.07, 0.03)	-0.04 (-0.13, 0.05)
Animal protein	0.06 (-0.09, 0.21)	0.04 (-0.08, 0.15)	0.01 (-0.08, 0.10)	-10.8 (-26.1, 4.50)	-0.02 (-0.11, 0.07)	0.04 (-0.04, 0.12)	-0.02 (-0.11, 0.06)	0.04 (-0.04, 0.12)	-0.02 (-0.11, 0.06)	0.04 (-0.04, 0.12)	-0.03 (-0.12, 0.06)	0 (-0.05, 0.05)	0 (-0.05, 0.05)	-0.03 (-0.12, 0.06)
Plant fat	0.01 (-0.14, 0.17)	0.01 (-0.11, 0.13)	0.02 (-0.08, 0.11)	-10.8 (-26.3, 4.70)	-0.03 (-0.12, 0.06)	0.02 (-0.07, 0.1)	-0.03 (-0.12, 0.06)	0.01 (-0.07, 0.09)	-0.03 (-0.12, 0.06)	0.01 (-0.07, 0.09)	0 (-0.09, 0.09)	-0.03 (-0.08, 0.02)	-0.03 (-0.08, 0.03)	0 (-0.09, 0.09)
Animal fat	0.07 (-0.08, 0.21)	0.05 (-0.06, 0.17)	0.03 (-0.06, 0.12)	-10.65 (-25.8, 4.50)	-0.01 (-0.1, 0.08)	0.03 (-0.05, 0.11)	-0.01 (-0.1, 0.08)	0.03 (-0.05, 0.11)	-0.01 (-0.1, 0.08)	0.03 (-0.05, 0.11)	0 (-0.09, 0.09)	-0.02 (-0.07, 0.03)	-0.02 (-0.07, 0.03)	0 (-0.09, 0.09)
Carbohydrates	-0.11 (-0.26, 0.04)	-0.06 (-0.17, 0.06)	-0.02 (-0.11, 0.07)	10.7 (-4.54, 25.9)	0.02 (-0.07, 0.1)	-0.01 (-0.1, 0.07)	0.02 (-0.07, 0.1)	-0.01 (-0.09, 0.07)	0.02 (-0.07, 0.1)	-0.01 (-0.09, 0.07)	0.01 (-0.07, 0.10)	-0.02 (-0.07, 0.03)	-0.02 (-0.07, 0.03)	0.01 (-0.08, 0.10)
Fiber	0 (-0.15, 0.15)	-0.02 (-0.13, 0.1)	-0.02 (-0.11, 0.07)	1.3 (-14.2, 16.8)	-0.05 (-0.14, 0.03)	0.05 (-0.04, 0.13)	-0.05 (-0.14, 0.03)	0.03 (-0.05, 0.11)	-0.05 (-0.14, 0.03)	0.03 (-0.04, 0.11)	-0.01 (-0.1, 0.08)	-0.02 (-0.07, 0.03)	-0.02 (-0.07, 0.03)	-0.01 (-0.10, 0.08)
**Fatty acids**														
SFAs	-0.01 (-0.15, 0.14)	-0.04 (-0.15, 0.07)	-0.05 (-0.13, 0.04)	-10.0 (-24.8, 4.70)	0.04 (-0.05, 0.12)	-0.01 (-0.09, 0.07)	0.04 (-0.05, 0.12)	0 (-0.08, 0.08)	0.04 (-0.05, 0.12)	0 (-0.07, 0.08)	0.02 (-0.06, 0.10)	-0.02 (-0.07, 0.03)	-0.02 (-0.07, 0.03)	0.02 (-0.07, 0.11)
MUFAs	-0.03 (-0.19, 0.12)	-0.05 (-0.17, 0.06)	-0.05 (-0.14, 0.04)	-9.6 (-25.0, 5.90)	0 (-0.08, 0.09)	0.01 (-0.07, 0.1)	0 (-0.08, 0.09)	0.02 (-0.06, 0.1)	0 (-0.08, 0.09)	0.02 (-0.06, 0.10)	0.02 (-0.06, 0.11)	-0.01 (-0.06, 0.04)	-0.01 (-0.06, 0.04)	0.03 (-0.06, 0.12)
PUFAs	0.10 (-0.05, 0.26)	0.05 (-0.07, 0.17)	0.01 (-0.08, 0.11)	-13.6 (-29.4, 2.30)	-0.02 (-0.11, 0.07)	0.05 (-0.03, 0.14)	-0.02 (-0.11, 0.07)	0.06 (-0.02, 0.14)	-0.02 (-0.11, 0.07)	0.06 (-0.02, 0.14)	-0.08 (-0.17, 0.01)	-0.01 (-0.06, 0.04)	-0.01 (-0.06, 0.04)	-0.08 (-0.17, 0.01)

*F/B* Firmicutes/Bacteroidetes, *SFAs* saturated fatty acids, *MUFAs* monounsaturated fatty acids, *PUFAs* polyunsaturated fatty acids

^a^Alpha-diversity indices are log-transformed

^b^Adjusted for age, sex, family history of colorectal cancer, smoking status, alcohol consumption, body mass index, American Joint Committee on Cancer stage, neoadjuvant therapy, and comorbidity

#### Linear discriminant analysis effect size

**Fig. 5:**
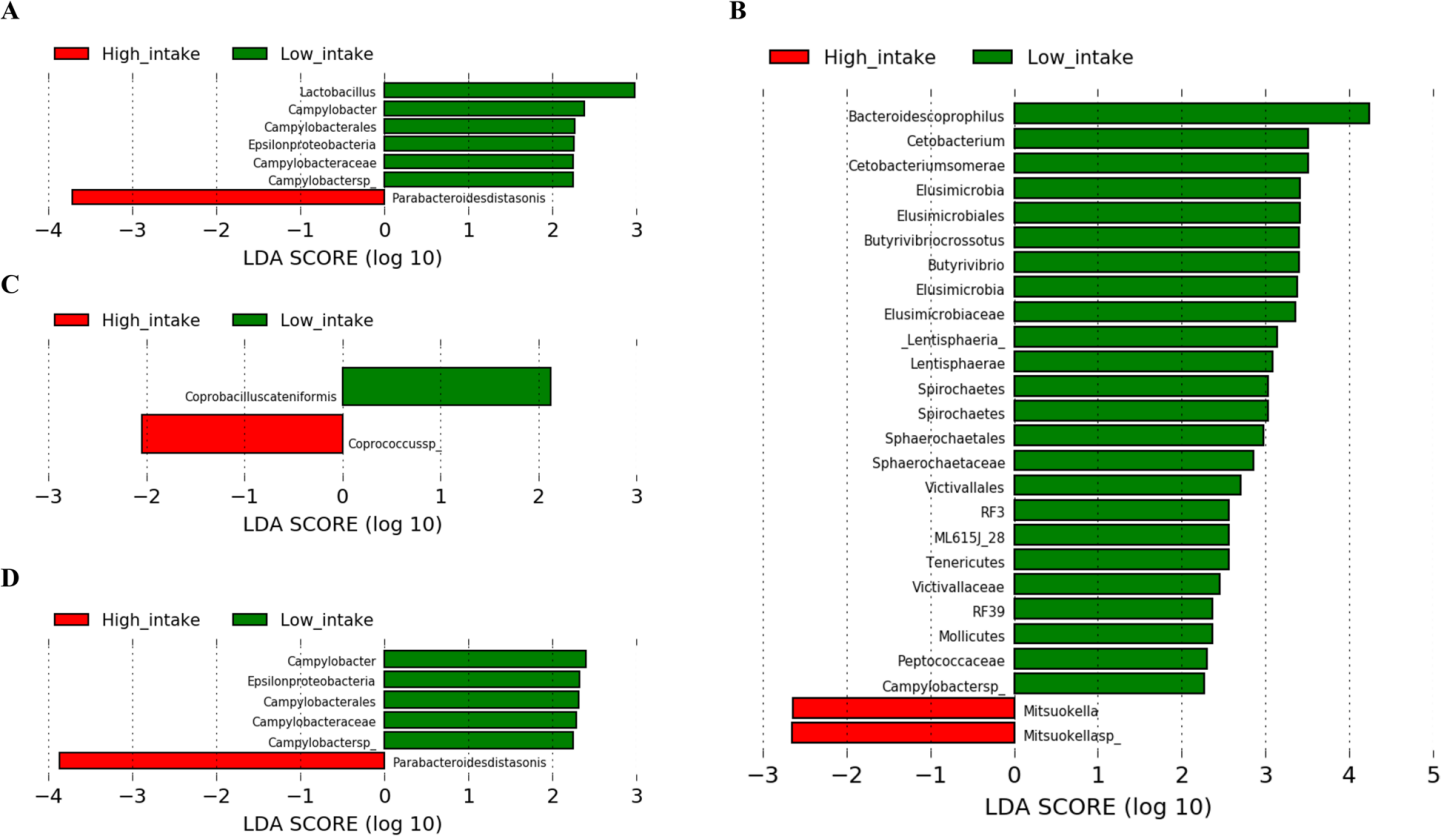
Linear discriminant analysis effect size analysis to identify enriched bacteria according to low and high intake groups of (**A**) sugars and sweets, (**B**) legumes, (**C**) mushrooms, (**D**) eggs, (**E**) oils and fats, (**F**) plant fat, (**G**) carbohydrate, and (**H**) monounsaturated fatty acids

**Fig. 6:**
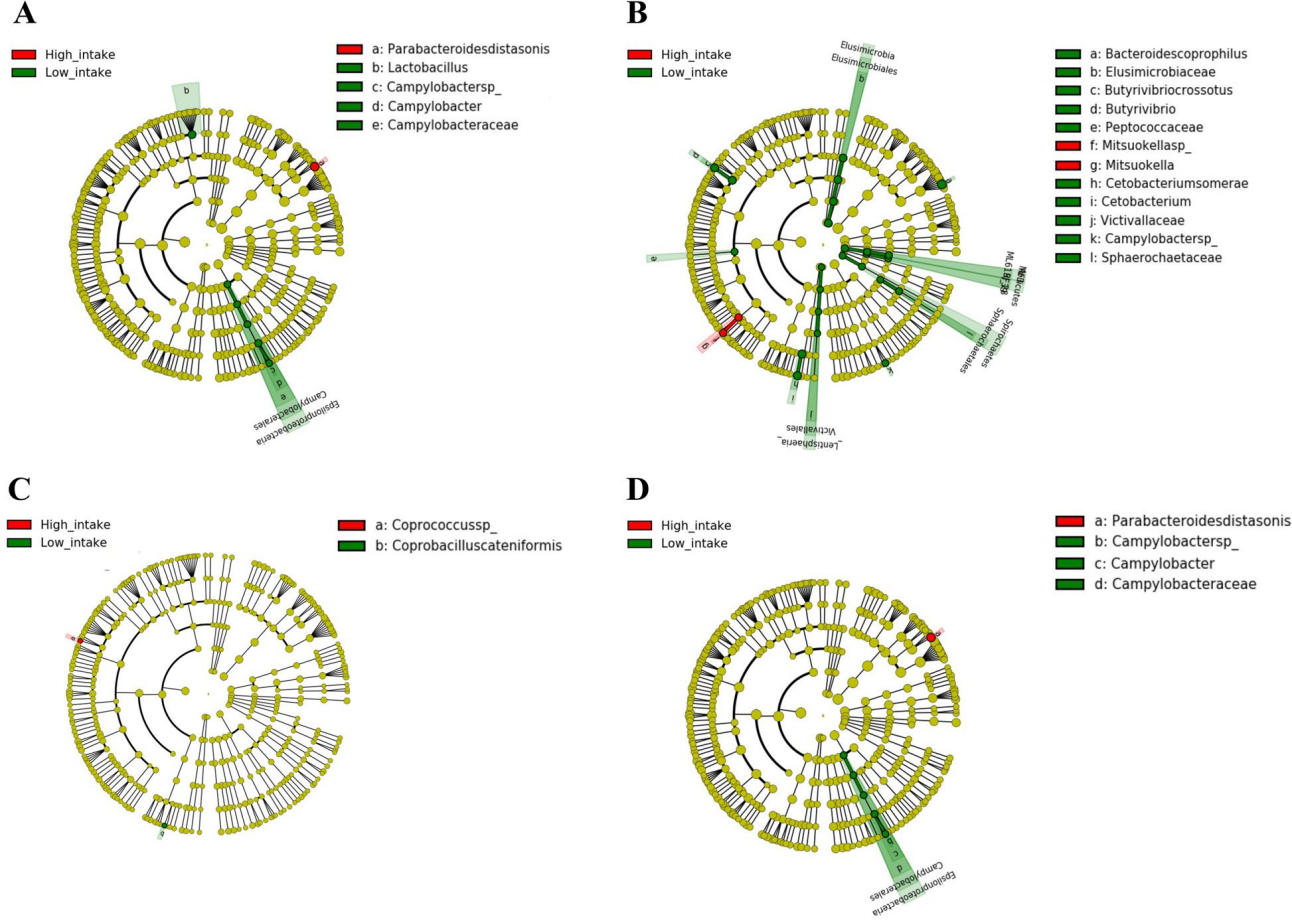
Cladogram of enriched bacteria according to low and high intake groups of (**A**) sugars and sweets, (**B**) legumes, (**C**) eggs, and (**D**) oils and fats

We performed the LEfSe analysis and constructed a cladogram to identify the phylogenetically enriched taxa in the low and high diet consumption groups. Of the 16 food groups, two dietary patterns, eight macronutrients, and three fatty acids, phylogenetically enriched taxa were identified according to the low and high intake of sugars and sweets, legumes, eggs, and oils and fats (Figs. 5A-D and 6A-D). In addition, enriched taxa were identified among patients in the low intake group of mushrooms (*Alistipes indistinctus*), plant fat (*Actinobacteria*), and carbohydrates (*Bacteroides fragilis*), and those in the high intake group of monounsaturated fatty acids (MUFAs) (*Clostridium symbiosum*) only (data not shown, LDA > 2.0, *p* < 0.01).

## Discussion

This is the first study to elucidate both the within-sample diversity and individual components of the gut microbial community in association with dietary features of a cohort of Korean CRC patients. We carried out a nutrition-wide association study on the effect of consumption of 106 food items, 16 food groups, two dietary patterns, and 92 nutrients on the overall microbial diversity of species richness and/or evenness and the abundance of 439 gut microbial taxa at different physiological levels. After multiple comparison adjustments, no significant correlations were observed between diet consumption and overall richness and/or evenness of the gut microbiota. However, we identified some bacteria that were phylogenetically enriched with higher or lower consumption of sugars and sweets, legumes, mushrooms, eggs, oils and fats, plant fat, carbohydrate, and MUFAs.

Previous studies have reported three PCA-derived dietary patterns in the Korean population [22, 34–40]; the traditional Korean diet is characterized by high intake of food items such as vegetables, seaweeds, fish, soy, and mushrooms [22]; the Western-style diet is characterized by high intake of different meat, fast food, and oil and sugar [22]; and the prudent pattern is characterized by high intake of fruits, milk, and dairy products, and low intake of refined grains [22]. In the present study, we identified only two dietary patterns (low fruit-vegetable and high fruit/low meat-poultry), with high intake of cereals and grains in both the patterns, but a distinction was observed in the factor loadings of vegetables, fruits, and meat and poultry; this could be due to the nature of data-driven methods, such as PCA, and the variation in habitual diets of CRC patients in comparison with that of the general population. Nevertheless, our PCA-derived dietary patterns are appropriate for CRC patients because the components of the two dietary patterns assist in CRC prevention [41, 42]. Furthermore, using clustering methods, we could classify study participants into separate dietary behavior groups and examine differences in their microbiome structure.

Our results for enterotypes are partially comparable to those of previous studies. In a cohort of 1,199 Korean adults, three enterotypes were identified, namely *Bacteroidaceae*, *Prevotellaceae*, and *Ruminococcaceae* [19]. In another cohort of 222 healthy Koreans, enterotypes including Bacteroidetes, *Prevotella*, and *Ruminococcus* were identified [18]. In the present study, we identified *Rikenellaceae* and *Alistipes* enterotypes instead of *Ruminococcaceae* and *Ruminococcus* at the family and genus levels, respectively. We also found that the main enterotypes did not separate from each other [18, 19], except when Bacteroidetes and *Alistipes* were combined into a single enterotype. Our results, therefore, suggest a higher abundance of *Alistipes* and its family compared to that of *Ruminococcus* and its family in CRC patients. This finding was in line with the results of previous studies reporting a higher abundance of *Ruminococcaceae* in healthy controls than in CRC tumor samples [43, 44]; the elevated abundance of *Rikenellaceae* in mucosa colon cancer patients compared to that in controls has also been reported [45]. Similarly, an overrepresentation of *Alistipes* in CRC patients and *Ruminococcus* in healthy controls was reported [46], which could explain our findings.

Non-toxigenic *Bacteroides fragilis* is not harmful to the intestinal tract, but another class called enterotoxigenic *Bacteroides fragilis* produces toxins, which may trigger the development of advanced CRC through the dysfunction of the intestinal immune system [11, 47, 48]. Nevertheless, a significantly lower 3-year overall survival and disease-free survival among those with a high abundance of *Bacteroides fragilis* than in the low-abundance group was observed in a pilot study of 180 CRC patients [49]. Approximately 48% fat and 39% lean are present in unprocessed pork (belly), mainly consisting of MUFAs (47%) and saturated fatty acids (36%) [50], which can promote or inhibit the outer membrane vesicles of *Bacteroides fragilis* in a fatty-acid-chain-length- and dose-dependent manner [51]. Furthermore, palmitoleic and palmitic acids exert an inhibitory effect on the growth of *Bacteroides fragilis* at low concentrations [51].

*Clostridium symbiosum*, which is involved in the butyrate-producing pathway [52], is postulated to activate protein synthesis in the local gut epithelium and enhance the development of carcinogenesis [53]. *Clostridium symbiosum* abundance has been reported to cause bacteremia in CRC patients, and noninvasive methods, such as fecal immunochemical test and carcinoembryonic antigen test, revealed an improvement in the efficacy of early CRC diagnosis [53, 54]. Although acid amines act as substrates by *Clostridium sp*. [55], the mechanism by which the consumption of amines was related to the decreased abundance of *Clostridium symbiosum* needs to be further elucidated.

Recent studies have reported the contribution of gut microbiota to the progression of CRC [11, 56]. Among them, *Fucobacterium nucleatum* is mostly associated with CpG island methylator phenotype, microsatellite instability, and *BRAF*, *KRAS*, *TP53*, *CHD7*, and *CHD8* mutations, which are suggested to predispose mortality related to CRC and worse clinical outcomes [57–59]. *F. nucleatum* was enriched in CRC patients with or without chemotherapy treatment and depleted in healthy or postoperative individuals [60]. In contrast, *Prevotella* and *Bacteroides* co-abundance groups and *Faecalibacterium prausnitzii* were found to be associated with better survival outcomes [61]. However, our study failed to detect any dietary factors affecting the relative abundance of *Fucobacterium nucleatum* and *Faecalibacterium sp.*

In Korea, edible seaweeds exist in water-containing or dried forms [62, 63]. Despite the complexity of structural and storage polysaccharides according to taxonomically different seaweeds, polysaccharides are the most abundant bioactive compounds in seaweeds [64]. In the digestive system, polysaccharides are proteolytically fermented as SCFAs and other end-products [64, 65]. The levels of SCFAs and intestinal bacterial communities may, therefore, reflect the effects of polysaccharides on the gut microbiota [66]. In particular, several polysaccharides from green algae have been shown to decrease the abundance of *Rikenellaceae* and *Alistipes* in mice [66], which supports our findings.

Previous studies have consistently shown the association of coffee and tea with a healthy gut microbial community [67, 68]. A study on 147 healthy individuals revealed a higher abundance of *Bacteroides-Prevotella-Porphyromonas* in individuals who consumed more coffee than in those who consumed less coffee [68], which could be due to the polyphenols and caffeine in coffee beverages [68]. In a mouse model of metabolic syndrome, partial effects on improving the gut dysbiosis and disrupted plasma SCFA profile were reported for caffeine and chlorogenic acids [69]. A pilot trial revealed possible effects of caffeine and chlorogenic acids in rising *Bifidobacterium* in patients with non-alcoholic fatty liver and diabetes, although the increases were not significant [70]. Furthermore, polyphenols present in both green tea and black tea have been reported to exert inhibitory effects on α-amylase and α-glucosidase in the saliva and small intestine, which can result in residual carbohydrates in the large intestine, providing a substrate for SCFAs and energy for colonic epithelium and peripheral tissues [71]. On the contrary, the effect of high-, low-, and non-calorie sweeteners on the abundance of *Bacteroidetes* remains controversial due to the complex polyols in these beverages, which limits the establishment of directionality [72, 73]. Given that caffeine is one of the biologically active compounds in coffee, tea, and carbonated drinks [74, 75], we considered these food items into a single food group of total beverages in the present study. Despite the variation in nutritional compositions, we did not find any significant correlations between the consumption of coffee, green tea, and carbonated drinks with microbiome diversity and abundance.

Studies have observed a positive association of within-sample microbial diversity with dietary quality indices in healthy adults [76–78]. Notably, a Western-style diet was associated with lower microbial diversity, whereas a plant-based diet was associated with higher microbial diversity [79]. In the present study, we did not observe any significant association between dietary features and alpha-diversity indices. Therefore, we suggest a weaker association of within-sample microbial diversity with dietary intake in CRC patients than in healthy subjects.

However, several limitations of this study must be acknowledged. First, given the cross-sectional study design, we could not determine the causal relationship and evaluate the effect of the diet–microbiome association on CRC recurrence and prognosis. However, the bias regarding this temporal relationship was minimized by assessing the average of habitual diets for the year prior to the date of fecal sample collection. Since our study population comprised Korean CRC patients in a hospital in Seoul, our findings might not be generalizable to other populations. Second, the possible measurement error and recall bias in using the FFQ for dietary assessment need to be addressed. However, the validated and reproducible FFQ for the Korean population was administered by well-trained staff, which minimized the risk of inaccurate collection of information [20, 80]. Third, there could be residual confounding due to the lack of information. In general, probiotics are introduced with beneficial functions by restoring the composition of the gut microbiome, whereas antibiotics may decrease the population of several bacteria [81, 82]. Besides, CRC patients were reported to commonly face with mental health conditions such as anxiety (1.6%-57%) and depression (1.0%-47.2%) [83]. In these conditions, there was a reduction of SCFA producing bacteria which can contribute to the gut permeability and systemic inflammation [84]. Thus, further studies may take the account for prebiotic and antibiotic use and mental health in the diet-microbiome interaction among CRC patients. Finally, by obtaining fecal samples at a single timepoint prior to the operation, we could not take into account the variability and stability of the microbial community at different timepoints. Although diet consumption accounted for a relatively small proportion of microbial variation at the population level [85–88], changing habitual diets might contribute to the modification of microbial composition at the individual level [85, 89–93]. Therefore, dense longitudinal studies are required to further elucidate personalized diet–microbiome relationships in CRC patients.

## Conclusion

In summary, our data provide comprehensive evidence for diet-microbe interactions in CRC patients. Although the dietary features were not associated with within-sample diversity, we identified several bacteria that were phylogenetically enriched according to the consumption of several food items, food groups, dietary patterns, and nutrients. Additional research is needed to understand the mechanisms underlying these observations as well as their significance in CRC prognosis.

## Supplementary Information

**Additional file 1: Figure S1. **Scree plot for variance of food groups explained by dietary patterns. **Figure S2.** Gaussian graphical model networks for pairwise correlations of relative abundances of phyla in (A) low fruit-vegetable and (B) high fruit/low meat-poultry groups. Nodes reflect phylum, and edges reflect the conditional dependencies between phyla. The size of the circles is proportional to the mean relative abundance of the corresponding phylum. Green lines show positive partial correlations, and red lines show negative partial correlations. The thickness of edges represents the strength of correlations. **Figure S3.** Gaussian graphical model networks for pairwise correlations of relative abundances of classes in (A) low fruit-vegetable and (B) high fruit/low meat-poultry dietary groups. Nodes reflect phylum, and edges reflect the conditional dependencies between classes. The size of the circles is proportional to the mean relative abundance of the corresponding class. Green lines show positive partial correlations, and red lines show negative partial correlations. The thickness of edges represents the strength of correlations. **Figure S4.** Gaussian graphical model networks for pairwise correlations of relative abundances of orders in A) low fruit-vegetable and (B) high fruit/low meat-poultry dietary groups. Nodes reflect phylum, and edges reflect the conditional dependencies between classes. The size of the circles is proportional to the mean relative abundance of the corresponding order. Green lines show positive partial correlations, and red lines show negative partial correlations. The thickness of edges represents the strength of correlations. **Figure S5.** Gaussian graphical model networks for pairwise correlations of relative abundances of families in A) low fruit-vegetable and (B) high fruit/low meat-poultry dietary groups. Nodes reflect phylum, and edges reflect the conditional dependencies between families. The size of the circles is proportional to the mean relative abundance of the corresponding family. Green lines show positive partial correlations, and red lines show negative partial correlations. The thickness of edges represents the strength of correlations. **Figure S6.** Gaussian graphical model networks for pairwise correlations of relative abundances of genera in A) low fruit-vegetable and (B) high fruit/low meat-poultry dietary groups. Nodes reflect phylum, and edges reflect the conditional dependencies between genera. The size of the circles is proportional to the mean relative abundance of the corresponding genus. Green lines show positive partial correlations, and red lines show negative partial correlations. The thickness of edges represents the strength of correlations.

**Additional file 2: Table S1. **Daily diet consumption and Spearman correlation between dietary factors and microbial alpha-diversity. **Table S2.** Pairwise correlations for relative abundances of different phylogenetical levels in low fruit-vegetable and high fruit/low meat-poultry dietary groups. **Table S3.** Spearman correlation coefficients between dietary factors and relative abundance of microbial taxonomy. **Table S4.** Crude *p*-values for Spearman correlation between dietary factors and relative abundance of microbial taxonomy. **Table S5.** False discovery rate adjusted *p*-values for Spearman correlation between dietary factors and relative abundance of microbial taxonomy.

## Data Availability

The sequencing dataset supporting the conclusions of this article is available from the NCBI Sequence Read Archive (SRA) repository, [PRJNA797640, in https://www.ncbi.nlm.nih.gov/sra/PRJNA797640]. All other data are available from the corresponding author by reasonable request.

## Abbreviations

AJCC: American Joint Committee on Cancer
BMI: Body mass index
CRC: Colorectal cancer
FFQ: Food frequency questionnaire
GGM: Gaussian graphical model
LDA: Linear discriminant analysis
LEfSe: LDA of effect size
F/B: Firmicutes/Bacteroidetes
MUFA: Monounsaturated fatty acid
OTU: Operational taxonomic unit
PCA: Principal component analysis
PCoA: Principal coordinate analysis
SCFA: Short-chain fatty acid

## References

[1] Thursby E, Juge N (2017). Introduction to the human gut microbiota. Biochem J.

[2] Cheng Y, Ling Z, Li L (2020). The intestinal microbiota and colorectal cancer. Front Immunol.

[3] Fan Y, Pedersen O (2021). Gut microbiota in human metabolic health and disease. Nat Rev Microbiol.

[4] Nichols RG, Davenport ER (2021). The relationship between the gut microbiome and host gene expression: a review. Hum Genet.

[5] Salazar N, Gonzalez S, Nogacka AM, Rios-Covian D, Arboleya S, Gueimonde M, Reyes-Gavilan CGL (2020). Microbiome: effects of ageing and diet. Curr Issues Mol Biol.

[6] Wang J, Chen WD, Wang YD (2020). The relationship between gut microbiota and inflammatory diseases: the role of macrophages. Front Microbiol.

[7] Sanchez-Alcoholado L, Ramos-Molina B, Otero A, Laborda-Illanes A, Ordonez R, Medina JA, Gomez-Millan J, Queipo-Ortuno MI. The role of the gut microbiome in colorectal cancer development and therapy response. Cancers (Basel). 2020;12(6):1406. 10.3390/cancers12061406 PMC7352899 32486066

[8] Durack J, Lynch SV (2019). The gut microbiome: relationships with disease and opportunities for therapy. J Exp Med.

[9] Ternes D, Karta J, Tsenkova M, Wilmes P, Haan S, Letellier E (2020). Microbiome in colorectal cancer: how to get from meta-omics to mechanism?. Trends Microbiol.

[10] Saus E, Iraola-Guzman S, Willis JR, Brunet-Vega A, Gabaldon T (2019). Microbiome and colorectal cancer: Roles in carcinogenesis and clinical potential. Mol Aspects Med.

[11] Montalban-Arques A, Scharl M (2019). Intestinal microbiota and colorectal carcinoma: Implications for pathogenesis, diagnosis, and therapy. EBioMedicine.

[12] Jin Y, Liu Y, Zhao L, Zhao F, Feng J, Li S, Chen H, Sun J, Zhu B, Geng R (2019). Gut microbiota in patients after surgical treatment for colorectal cancer. Environ Microbiol.

[13] Valdes AM, Walter J, Segal E, Spector TD (2018). Role of the gut microbiota in nutrition and health. BMJ.

[14] Song M, Chan AT (2019). Environmental factors, gut microbiota, and colorectal cancer prevention. Clin Gastroenterol Hepatol.

[15] Tomova A, Bukovsky I, Rembert E, Yonas W, Alwarith J, Barnard ND, Kahleova H (2019). The effects of vegetarian and vegan diets on gut microbiota. Front Nutr.

[16] Ma B, Wang Y, Ye S, Liu S, Stirling E, Gilbert JA, Faust K, Knight R, Jansson JK, Cardona C (2020). Earth microbial co-occurrence network reveals interconnection pattern across microbiomes. Microbiome.

[17] Kim SH, Kim MS, Lee MS, Park YS, Lee HJ, Kang S, Lee HS, Lee KE, Yang HJ, Kim MJ (2016). Korean diet: characteristics and historical background. J Ethn Foods.

[18] Noh H, Jang HH, Kim G, Zouiouich S, Cho SY, Kim HJ, Kim J, Choe JS, Gunter MJ, Ferrari P, et al. Taxonomic composition and diversity of the gut microbiota in relation to habitual dietary intake in Korean adults. Nutrients. 2021;13(2):366. 10.3390/nu13020366 PMC7912254 33530330

[19] Wu X, Unno T, Kang S, Park S. A Korean-style balanced diet Has a potential connection with Ruminococcaceae enterotype and reduction of metabolic syndrome incidence in Korean adults. Nutrients. 2021;13(2):495. 10.3390/nu13020495 PMC7913599 33546299

[20] Ahn Y, Kwon E, Shim JE, Park MK, Joo Y, Kimm K, Park C, Kim DH (2007). Validation and reproducibility of food frequency questionnaire for Korean genome epidemiologic study. Eur J Clin Nutr.

[21] Brown CC, Kipnis V, Freedman LS, Hartman AM, Schatzkin A, Wacholder S (1994). Energy adjustment methods for nutritional epidemiology: the effect of categorization. Am J Epidemiol.

[22] Park Y, Lee J, Oh JH, Shin A, Kim J (2016). Dietary patterns and colorectal cancer risk in a Korean population: A case-control study. Medicine (Baltimore).

[23] Package ‘factoextra’: Extract and visualize the results of multivariate data analyses [https://cloud.r-project.org/web/packages/factoextra/factoextra.pdf]

[24] Package ‘vegan’: Community ecology package [https://cran.r-project.org/web/packages/vegan/vegan.pdf]

[25] Chen B, He X, Pan B, Zou X, You N (2021). Comparison of beta diversity measures in clustering the high-dimensional microbial data. PLoS ONE.

[26] Package ‘cluster’: "Finding Groups in Data": Cluster Analysis Extended Rousseeuw et al. [https://cran.r-project.org/web/packages/cluster/cluster.pdf]

[27] Xia Y, Sun J, Chen DG. Statistical Analysis of Microbiome Data with R. Springer Nature Singapore. 2018. p. 167–90.

[28] Package ‘zCompositions’: Treatment of zeros, left-censored and missing values in compositional data sets [https://cran.r-project.org/web/packages/zCompositions/zCompositions.pdf]

[29] Iqbal K, Buijsse B, Wirth J, Schulze MB, Floegel A, Boeing H (2016). Gaussian graphical models identify networks of dietary intake in a German adult population. J Nutr.

[30] Iqbal K, Schwingshackl L, Floegel A, Schwedhelm C, Stelmach-Mardas M, Wittenbecher C, Galbete C, Knuppel S, Schulze MB, Boeing H (2019). Gaussian graphical models identified food intake networks and risk of type 2 diabetes, CVD, and cancer in the EPIC-Potsdam study. Eur J Nutr.

[31] Package ‘glasso’: Graphical lasso: estimation of Gaussian graphical models [https://cran.r-project.org/web/packages/glasso/glasso.pdf]

[32] Stadler N, Mukherjee S (2015). Multivariate gene-set testing based on graphical models. Biostatistics.

[33] Package ‘nethet’: A Bioconductor package for investigation of network heterogeneity from high-dimensional data [https://www.bioconductor.org/packages/release/bioc/vignettes/nethet/inst/doc/nethet.pdf]

[34] Cho YA, Shin A, Kim J (2011). Dietary patterns are associated with body mass index in a Korean population. J Am Diet Assoc.

[35] Choi JH, Woo HD, Lee JH, Kim J (2015). Dietary patterns and risk for metabolic syndrome in Korean women: a cross-sectional study. Medicine (Baltimore).

[36] Woo HD, Shin A, Kim J (2014). Dietary patterns of Korean adults and the prevalence of metabolic syndrome: a cross-sectional study. PLoS One.

[37] Wie GA, Cho YA, Kang HH, Ryu KA, Yoo MK, Kim J, Shin S, Chun OK, Joung H (2017). Identification of major dietary patterns in Korean adults and their association with cancer risk in the Cancer Screening Examination Cohort. Eur J Clin Nutr.

[38] Kim SA, Joung H, Shin S (2019). Dietary pattern, dietary total antioxidant capacity, and dyslipidemia in Korean adults. Nutr J.

[39] Lim JH, Lee YS, Chang HC, Moon MK, Song Y (2011). Association between dietary patterns and blood lipid profiles in Korean adults with type 2 diabetes. J Korean Med Sci.

[40] Cho YA, Kim J, Shin A, Park KS, Ro J (2010). Dietary patterns and breast cancer risk in Korean women. Nutr Cancer.

[41] Mejborn H, Moller SP, Thygesen LC, Biltoft-Jensen A. Dietary intake of red meat, processed meat, and poultry and risk of colorectal cancer and all-cause mortality in the context of dietary guideline compliance. Nutrients. 2020;13(1):32. 10.3390/nu13010032 PMC7823645 33374887

[42] Kunzmann AT, Coleman HG, Huang WY, Cantwell MM, Kitahara CM, Berndt SI (2016). Fruit and vegetable intakes and risk of colorectal cancer and incident and recurrent adenomas in the PLCO cancer screening trial. Int J Cancer.

[43] Huybrechts I, Zouiouich S, Loobuyck A, Vandenbulcke Z, Vogtmann E, Pisanu S, Iguacel I, Scalbert A, Indave I, Smelov V (2020). The Human Microbiome in Relation to Cancer Risk: A Systematic Review of Epidemiologic Studies. Cancer Epidemiol Biomarkers Prev.

[44] Chen W, Liu F, Ling Z, Tong X, Xiang C (2012). Human intestinal lumen and mucosa-associated microbiota in patients with colorectal cancer. PLoS ONE.

[45] Hibberd AA, Lyra A, Ouwehand AC, Rolny P, Lindegren H, Cedgard L, Wettergren Y (2017). Intestinal microbiota is altered in patients with colon cancer and modified by probiotic intervention. BMJ Open Gastroenterol.

[46] Feng Q, Liang S, Jia H, Stadlmayr A, Tang L, Lan Z, Zhang D, Xia H, Xu X, Jie Z (2015). Gut microbiome development along the colorectal adenoma-carcinoma sequence. Nat Commun.

[47] Cheng WT, Kantilal HK, Davamani F (2020). The mechanism of Bacteroides fragilis toxin contributes to colon cancer formation. Malays J Med Sci.

[48] Haghi F, Goli E, Mirzaei B, Zeighami H (2019). The association between fecal enterotoxigenic B. fragilis with colorectal cancer. BMC Cancer..

[49] Wei Z, Cao S, Liu S, Yao Z, Sun T, Li Y, Li J, Zhang D, Zhou Y (2016). Could gut microbiota serve as prognostic biomarker associated with colorectal cancer patients' survival? A pilot study on relevant mechanism. Oncotarget.

[50] Choe JH, Yang HS, Lee SH, Go GW (2015). Characteristics of pork belly consumption in South Korea and their health implication. J Anim Sci Technol.

[51] Mirjafari Tafti ZS, Moshiri A, Ettehad Marvasti F, Tarashi S, Sadati Khalili SF, Motahhary A, Fateh A, Vaziri F, Ahmadi Badi S, Siadat SD (2019). The effect of saturated and unsaturated fatty acids on the production of outer membrane vesicles from Bacteroides fragilis and Bacteroides thetaiotaomicron. Gastroenterol Hepatol Bed Bench.

[52] Louis P, Flint HJ (2017). Formation of propionate and butyrate by the human colonic microbiota. Environ Microbiol.

[53] Xie YH, Gao QY, Cai GX, Sun XM, Sun XM, Zou TH, Chen HM, Yu SY, Qiu YW, Gu WQ (2017). Fecal Clostridium symbiosum for noninvasive detection of early and advanced colorectal cancer: test and validation studies. EBioMedicine.

[54] Chenard T, Malick M, Dube J, Masse E (2020). The influence of blood on the human gut microbiome. BMC Microbiol.

[55] Guo P, Zhang K, Ma X, He P (2020). Clostridium species as probiotics: potentials and challenges. J Anim Sci Biotechnol.

[56] Wong SH, Yu J (2019). Gut microbiota in colorectal cancer: mechanisms of action and clinical applications. Nat Rev Gastroenterol Hepatol.

[57] Chen Y, Yang Y, Gu J (2020). Clinical implications of the associations between intestinal microbiome and colorectal cancer progression. Cancer Manag Res.

[58] Saito K, Koido S, Odamaki T, Kajihara M, Kato K, Horiuchi S, Adachi S, Arakawa H, Yoshida S, Akasu T (2019). Metagenomic analyses of the gut microbiota associated with colorectal adenoma. PLoS ONE.

[59] Mima K, Nishihara R, Qian ZR, Cao Y, Sukawa Y, Nowak JA, Yang J, Dou R, Masugi Y, Song M (2016). Fusobacterium nucleatum in colorectal carcinoma tissue and patient prognosis. Gut.

[60] Deng X, Li Z, Li G, Li B, Jin X, Lyu G (2018). Comparison of microbiota in patients treated by surgery or chemotherapy by 16S rRNA sequencing reveals potential biomarkers for colorectal cancer therapy. Front Microbiol.

[61] Lauka L, Reitano E, Carra MC, Gaiani F, Gavriilidis P, Brunetti F (2019). de'Angelis GL, Sobhani I, de'Angelis N: Role of the intestinal microbiome in colorectal cancer surgery outcomes. World J Surg Oncol.

[62] Park JK, Woo HW, Kim MK, Shin J, Lee YH, Shin DH, Shin MH, Choi BY. Dietary iodine, seaweed consumption, and incidence risk of metabolic syndrome among postmenopausal women: a prospective analysis of the Korean Multi-Rural Communities Cohort Study (MRCohort). Eur J Nutr. 2020;60(1):135–46. 10.1007/s00394-020-02225-0 32211932

[63] Kim J, Lee J, Oh JH, Chang HJ, Sohn DK, Shin A, Kim J (2020). Associations among dietary seaweed intake, c-MYC rs6983267 polymorphism, and risk of colorectal cancer in a Korean population: a case-control study. Eur J Nutr.

[64] Charoensiddhi S, Conlon MA, Franco CMM, Zhang W (2017). The development of seaweed-derived bioactive compounds for use as prebiotics and nutraceuticals using enzyme technologies. Trends Food Sci Technol.

[65] Shang Q, Jiang H, Cai C, Hao J, Li G, Yu G (2018). Gut microbiota fermentation of marine polysaccharides and its effects on intestinal ecology: An overview. Carbohydr Polym.

[66] Lopez-Santamarina A, Miranda JM, Mondragon ADC, Lamas A, Cardelle-Cobas A, Franco CM, Cepeda A. Potential use of marine seaweeds as prebiotics: a review. Molecules. 2020;25(4):1004. 10.3390/molecules25041004 PMC7070434 32102343

[67] Bond T, Derbyshire E. Tea compounds and the gut microbiome: findings from trials and mechanistic studies. Nutrients. 2019;11(10):2364. 10.3390/nu11102364 PMC6835862 31623411

[68] Gonzalez S, Salazar N, Ruiz-Saavedra S, Gomez-Martin M, de Los Reyes-Gavilan CG, Gueimonde M. Long-term coffee consumption is associated with fecal microbial composition in humans. Nutrients. 2020;12(5):1287. 10.3390/nu12051287 PMC7282261 32369976

[69] Nishitsuji K, Watanabe S, Xiao J, Nagatomo R, Ogawa H, Tsunematsu T, Umemoto H, Morimoto Y, Akatsu H, Inoue K (2018). Effect of coffee or coffee components on gut microbiome and short-chain fatty acids in a mouse model of metabolic syndrome. Sci Rep.

[70] Mansour A, Mohajeri-Tehrani MR, Karimi S, Sanginabadi M, Poustchi H, Enayati S, Asgarbeik S, Nasrollahzadeh J, Hekmatdoost A (2020). Short term effects of coffee components consumption on gut microbiota in patients with non-alcoholic fatty liver and diabetes: A pilot randomized placebo-controlled, clinical trial. EXCLI J.

[71] Henning SM, Yang J, Hsu M, Lee RP, Grojean EM, Ly A, Tseng CH, Heber D, Li Z (2018). Decaffeinated green and black tea polyphenols decrease weight gain and alter microbiome populations and function in diet-induced obese mice. Eur J Nutr.

[72] Plaza-Diaz J, Pastor-Villaescusa B, Rueda-Robles A, Abadia-Molina F, Ruiz-Ojeda FJ. Plausible biological interactions of low- and non-calorie sweeteners with the intestinal microbiota: an update of recent studies. Nutrients. 2020;12(4):1153. 10.3390/nu12041153 PMC7231174 32326137

[73] Ruiz-Ojeda FJ, Plaza-Diaz J, Saez-Lara MJ, Gil A (2019). Effects of sweeteners on the gut microbiota: a review of experimental studies and clinical trials. Adv Nutr..

[74] Reyes CM, Cornelis MC. Caffeine in the diet: country-level consumption and guidelines. Nutrients. 2018;10(11):1172. 10.3390/nu10111772 PMC6266969 30445721

[75] McCusker RR, Goldberger BA, Cone EJ (2006). Caffeine content of energy drinks, carbonated sodas, and other beverages. J Anal Toxicol.

[76] Yu D, Nguyen SM, Yang Y, Xu W, Cai H, Wu J, Cai Q, Long J, Zheng W, Shu XO (2021). Long-term diet quality is associated with gut microbiome diversity and composition among urban Chinese adults. Am J Clin Nutr.

[77] Maskarinec G, Hullar MAJ, Monroe KR, Shepherd JA, Hunt J, Randolph TW, Wilkens LR, Boushey CJ, Le Marchand L, Lim U (2019). Fecal microbial diversity and structure are associated with diet quality in the multiethnic cohort adiposity phenotype study. J Nutr.

[78] Bowyer RCE, Jackson MA, Pallister T, Skinner J, Spector TD, Welch AA, Steves CJ (2018). Use of dietary indices to control for diet in human gut microbiota studies. Microbiome.

[79] Zhernakova A, Kurilshikov A, Bonder MJ, Tigchelaar EF, Schirmer M, Vatanen T, Mujagic Z, Vila AV, Falony G, Vieira-Silva S (2016). Population-based metagenomics analysis reveals markers for gut microbiome composition and diversity. Science.

[80] Kim J (2014). Cancer screenee cohort study of the National Cancer Center in South Korea. Epidemiol Health.

[81] Hemarajata P, Versalovic J (2013). Effects of probiotics on gut microbiota: mechanisms of intestinal immunomodulation and neuromodulation. Therap Adv Gastroenterol.

[82] Elvers KT, Wilson VJ, Hammond A, Duncan L, Huntley AL, Hay AD, van der Werf ET (2020). Antibiotic-induced changes in the human gut microbiota for the most commonly prescribed antibiotics in primary care in the UK: a systematic review. BMJ Open.

[83] Peng YN, Huang ML, Kao CH. Prevalence of Depression and Anxiety in Colorectal Cancer Patients: A Literature Review. Int J Environ Res Public Health. 2019;16(3):411. 10.3390/ijerph16030411 PMC6388369 30709020

[84] Halverson T, Alagiakrishnan K (2020). Gut microbes in neurocognitive and mental health disorders. Ann Med.

[85] Johnson AJ, Vangay P, Al-Ghalith GA, Hillmann BM, Ward TL, Shields-Cutler RR, Kim AD, Shmagel AK, Syed AN, Personalized Microbiome Class S (2019). Daily Sampling Reveals Personalized Diet-Microbiome Associations in Humans. Cell Host Microbe..

[86] Vangay P, Johnson AJ, Ward TL, Al-Ghalith GA, Shields-Cutler RR, Hillmann BM, Lucas SK, Beura LK, Thompson EA, Till LM (2018). US Immigration Westernizes the Human Gut Microbiome. Cell..

[87] Rothschild D, Weissbrod O, Barkan E, Kurilshikov A, Korem T, Zeevi D, Costea PI, Godneva A, Kalka IN, Bar N (2018). Environment dominates over host genetics in shaping human gut microbiota. Nature.

[88] Falony G, Joossens M, Vieira-Silva S, Wang J, Darzi Y, Faust K, Kurilshikov A, Bonder MJ, Valles-Colomer M, Vandeputte D (2016). Population-level analysis of gut microbiome variation. Science.

[89] Maier TV, Lucio M, Lee LH, VerBerkmoes NC, Brislawn CJ, Bernhardt J, Lamendella R, McDermott JE, Bergeron N, Heinzmann SS, et al. Impact of Dietary Resistant Starch on the Human Gut Microbiome, Metaproteome, and Metabolome. mBio. 2017;8(5):e01343–17. 10.1128/mBio.01343-17 PMC5646248 29042495

[90] Fukuyama J, Rumker L, Sankaran K, Jeganathan P, Dethlefsen L, Relman DA, Holmes SP (2017). Multidomain analyses of a longitudinal human microbiome intestinal cleanout perturbation experiment. PLoS Comput Biol.

[91] Flores GE, Caporaso JG, Henley JB, Rideout JR, Domogala D, Chase J, Leff JW, Vazquez-Baeza Y, Gonzalez A, Knight R (2014). Temporal variability is a personalized feature of the human microbiome. Genome Biol.

[92] David LA, Materna AC, Friedman J, Campos-Baptista MI, Blackburn MC, Perrotta A, Erdman SE, Alm EJ (2014). Host lifestyle affects human microbiota on daily timescales. Genome Biol.

[93] David LA, Maurice CF, Carmody RN, Gootenberg DB, Button JE, Wolfe BE, Ling AV, Devlin AS, Varma Y, Fischbach MA (2014). Diet rapidly and reproducibly alters the human gut microbiome. Nature.

