# Supplementary material for: Nutrition-wide association study of microbiome diversity and composition in colorectal cancer patients
Source: BMC Cancer. 2022 Jun 14;22:656. doi: 10.1186/s12885-022-09735-6 (PMC9199192; doi:10.1186/s12885-022-09735-6)
Supplement: Supplementary file 1 — Additional file 1: Figure S1. Scree plot for variance of food groups explained by dietary patterns. Figure S2. Gaussian graphical model networks for pairwise correlations of relative abundances of phyla in (A) low fruit-vegetable and (B) high fruit/low meat-poultry groups. Nodes reflect phylum, and edges reflect the conditional dependencies between phyla. The size of the circles is proportional to the mean relative abundance of the corresponding phylum. Green lines show positive partial correlations, and red lines show negative partial correlations. The thickness of edges represents the strength of correlations. Figure S3. Gaussian graphical model networks for pairwise correlations of relative abundances of classes in (A) low fruit-vegetable and (B) high fruit/low meat-poultry dietary groups. Nodes reflect phylum, and edges reflect the conditional dependencies between classes. The size of the circles is proportional to the mean relative abundance of the corresponding class. Green lines show positive partial correlations, and red lines show negative partial correlations. The thickness of edges represents the strength of correlations. Figure S4. Gaussian graphical model networks for pairwise correlations of relative abundances of orders in A) low fruit-vegetable and (B) high fruit/low meat-poultry dietary groups. Nodes reflect phylum, and edges reflect the conditional dependencies between classes. The size of the circles is proportional to the mean relative abundance of the corresponding order. Green lines show positive partial correlations, and red lines show negative partial correlations. The thickness of edges represents the strength of correlations. Figure S5. Gaussian graphical model networks for pairwise correlations of relative abundances of families in A) low fruit-vegetable and (B) high fruit/low meat-poultry dietary groups. Nodes reflect phylum, and edges reflect the conditional dependencies between families. The size of the circles is proportional to the me [file 12885_2022_9735_MOESM1_ESM.docx]

**Nutrition-wide Association Study of Microbiome Diversity and Composition**

**in Colorectal Cancer Patients**

**Authors and affiliations**

Tung Hoang ^a,b^, Min Jung Kim ^c,*^, Ji Won Park ^c^, Seung-Yong Jeong ^c^, Jeeyoo Lee ^a^, Aesun Shin ^a,b,d,*^

^a^ Department of Preventive Medicine, Seoul National University College of Medicine, Jongno-gu, Seoul 03080, South Korea

^b^ Integrated Major in Innovative Medical Science, Seoul National University College of Medicine, Jongno-gu, Seoul 03080, South Korea

^c^ Department of Surgery, Seoul National University College of Medicine, Jongno-gu, Seoul 03080, South Korea

^d^ Cancer Research Institute, Seoul National University College of Medicine, Jongno-gu, Seoul 03080, South Korea

**^*^ Corresponding authors**

Min Jung Kim, MD, PhD. Department of Surgery, Seoul National University College of Medicine, Jongno-gu, Seoul 03080, South Korea. Tel: +82-2-2072-7211. E-mail address: [surgeon.mjkim@gmail.com](mailto:surgeon.mjkim@gmail.com)

Aesun Shin, MD, PhD. Department of Preventive Medicine, Seoul National University College of Medicine, Jongno-gu, Seoul 03080, South Korea. Tel: +82-2-740-8331. E-mail: [shinaesun@snu.ac.kr](mailto:shinaesun@snu.ac.kr)

**
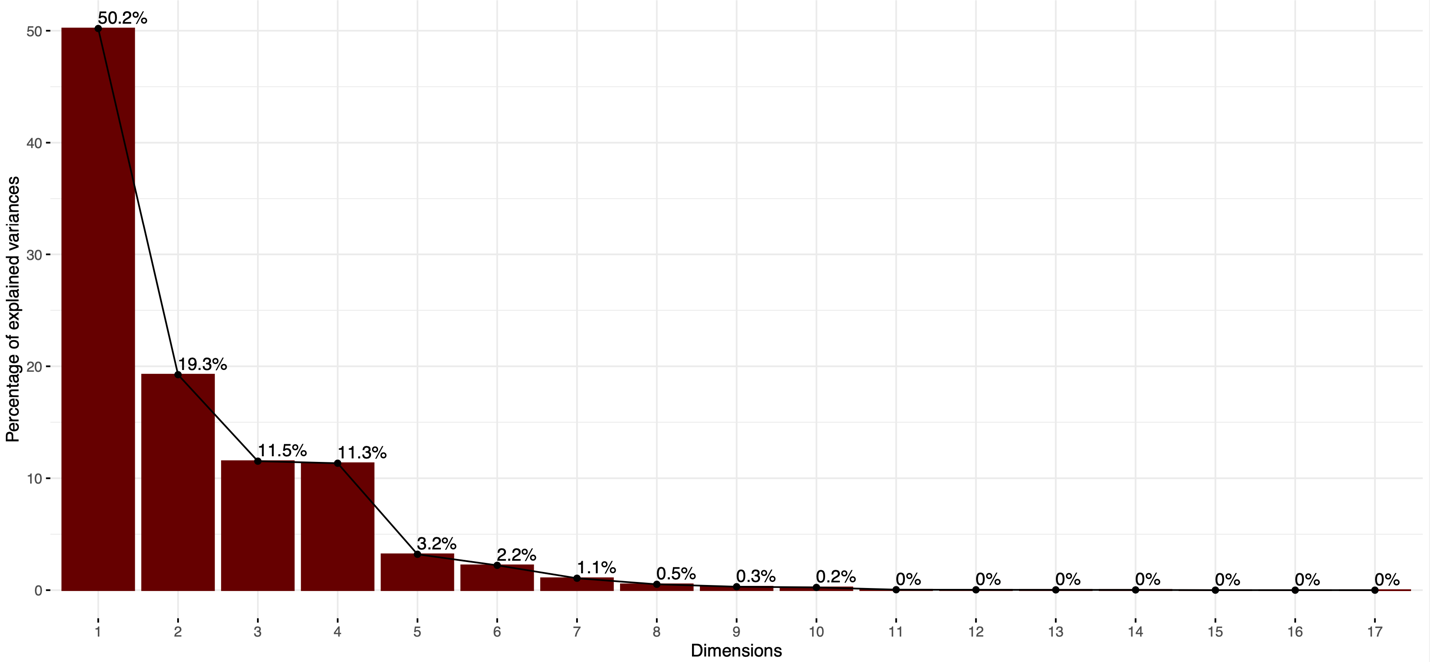
**

**Figure S1.** Scree plot for variance of food groups explained by dietary patterns.

**Figure S2.** Gaussian graphical model networks for pairwise correlations of relative abundances of phyla in (A) low fruit-vegetable and (B) high fruit/low meat-poultry groups. Nodes reflect phylum, and edges reflect the conditional dependencies between phyla. The size of the circles is proportional to the mean relative abundance of the corresponding phylum. Green lines show positive partial correlations, and red lines show negative partial correlations. The thickness of edges represents the strength of correlations.

**Figure S3.** Gaussian graphical model networks for pairwise correlations of relative abundances of classes in (A) low fruit-vegetable and (B) high fruit/low meat-poultry dietary groups. Nodes reflect phylum, and edges reflect the conditional dependencies between classes. The size of the circles is proportional to the mean relative abundance of the corresponding class. Green lines show positive partial correlations, and red lines show negative partial correlations. The thickness of edges represents the strength of correlations.

**Figure S4.** Gaussian graphical model networks for pairwise correlations of relative abundances of orders in A) low fruit-vegetable and (B) high fruit/low meat-poultry dietary groups. Nodes reflect phylum, and edges reflect the conditional dependencies between classes. The size of the circles is proportional to the mean relative abundance of the corresponding order. Green lines show positive partial correlations, and red lines show negative partial correlations. The thickness of edges represents the strength of correlations.

**Figure S5.** Gaussian graphical model networks for pairwise correlations of relative abundances of families in A) low fruit-vegetable and (B) high fruit/low meat-poultry dietary groups. Nodes reflect phylum, and edges reflect the conditional dependencies between families. The size of the circles is proportional to the mean relative abundance of the corresponding family. Green lines show positive partial correlations, and red lines show negative partial correlations. The thickness of edges represents the strength of correlations.

**Figure S6.** Gaussian graphical model networks for pairwise correlations of relative abundances of genera in A) low fruit-vegetable and (B) high fruit/low meat-poultry dietary groups. Nodes reflect phylum, and edges reflect the conditional dependencies between genera. The size of the circles is proportional to the mean relative abundance of the corresponding genus. Green lines show positive partial correlations, and red lines show negative partial correlations. The thickness of edges represents the strength of correlations.
